# Platelet-derived S100A9 contributes to endotheliopathy in alcohol-associated hepatitis

**DOI:** 10.1172/jci.insight.201114

**Published:** 2026-08-24

**Authors:** Fallyn Kirlin, Nima Fattahi, Rolando Garcia-Milian, Florine Collin, Weiwei Wang, Yohan Kim, Fabrice Lucien, Zhaoli Sun, TuKiet T. Lam, John Hwa, Yasuko Iwakiri, Matthew J. McConnell

**Affiliations:** 1 Section of Digestive Diseases,; 2 Bioinformatics Support Hub, Cushing/Whitney Medical Library, and; 3 Keck Mass Spectrometry & Proteomics Resource, Yale University School of Medicine, New Haven, Connecticut, USA.; 4 Department of Immunology and; 5 Department of Urology, Mayo Clinic, Rochester, Minnesota, USA.; 6 Department of Surgery, Johns Hopkins University School of Medicine, Baltimore, Maryland, USA.; 7 Department of Molecular Biophysics and Biochemistry, Yale University, New Haven, Connecticut, USA.; 8 Section of Cardiovascular Medicine, Yale University School of Medicine, New Haven, Connecticut, USA.

**Keywords:** Hematology, Hepatology, Endothelial cells, Hepatitis, Platelets

## Abstract

Alcohol-associated liver disease (ALD) is a growing global health concern, with alcohol-associated hepatitis (AH) leading to the highest morbidity and mortality. Available therapies are limited and often inadequate. Platelets contribute in a variety of ways to liver disease pathogenesis, but their role in AH remains largely unexplored. In this study, we addressed the hypothesis that platelets contribute to pathological inflammation in AH. Using patient samples and a multiomics approach, we found that platelets undergo proinflammatory transcriptomic and proteomic changes in AH, with 2 alarmins, S100A8 and S100A9, being among the top upregulated genes/proteins. Additionally, the abundance of platelet-derived microparticles containing S100A8 and S100A9 in AH patient plasma was increased and correlated with disease severity (assessed by model for end-stage liver disease sodium [MELD-Na]) and endotheliopathy (assessed by ICAM1, CXCL8, and vWF). We mechanistically linked S100A9 with endotheliopathy via crosstalk between primary human liver sinusoidal endothelial cells and primary human monocytes. We also demonstrated that IL-6 upregulates S100A9 in megakaryocytic cells in a JAK/STAT-dependent manner, modeling changes occurring in the bone marrow in patients with AH. Our studies establish proinflammatory platelets as important contributors to AH pathology. Moreover, antiplatelet agents — or, more specifically, S100A9 targeted drugs — are potential therapeutic strategies in AH.

## Abstract 2

Platelets upregulate inflammatory signals in alcohol-associated hepatitis that can contribute to liver inflammation. Platelets may also be a potential therapeutic target in this disease.

## Graphical abstract

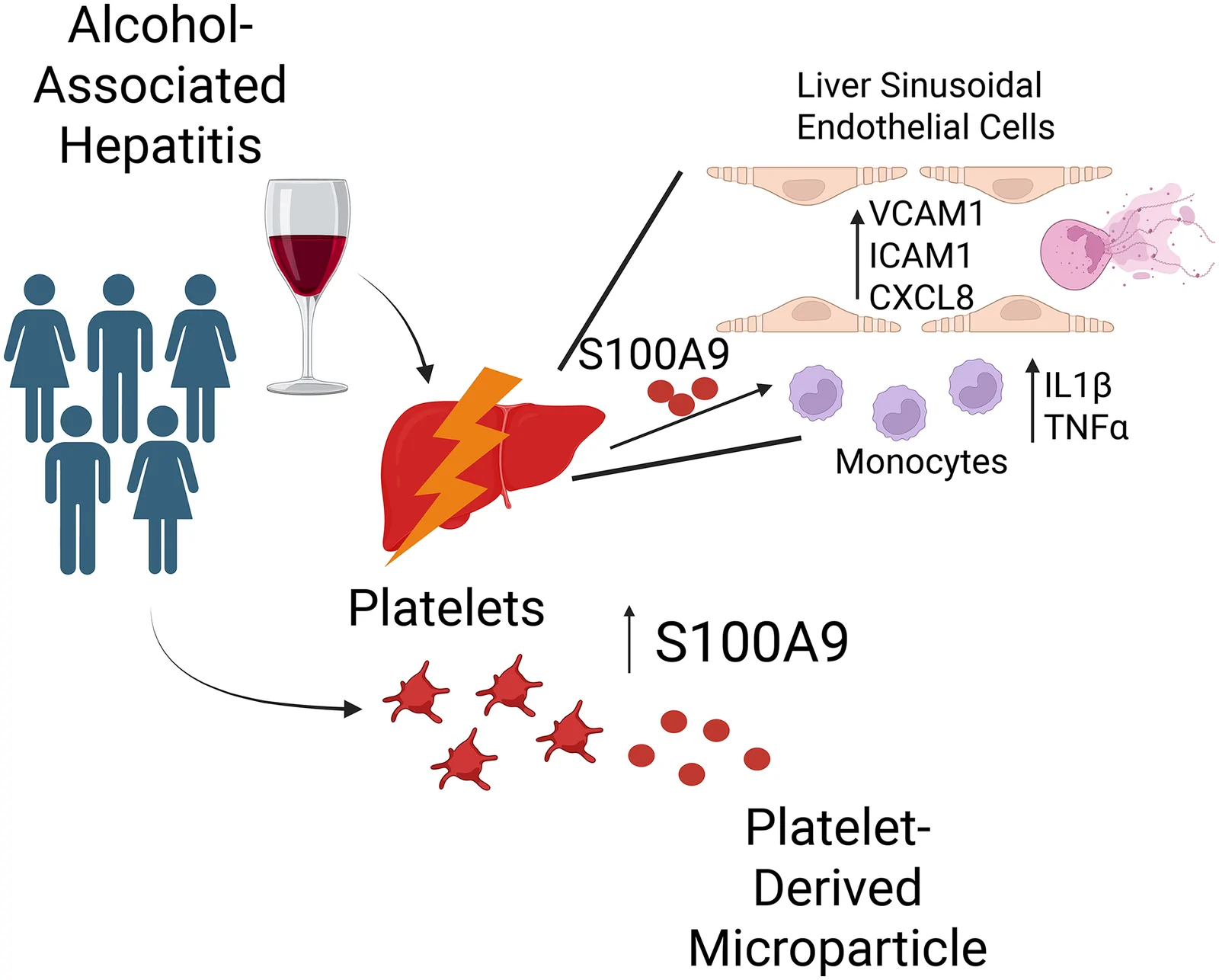

## Introduction

Alcohol-associated liver disease (ALD) is a pressing public health problem with a high burden of disease (1). Recent studies have shown that, in the wake of the COVID-19 pandemic, death from alcohol-associated hepatitis (AH), the most severe inflammatory form of ALD, has markedly increased (2). Severe AH has a very high mortality rate, reaching up to 20% in 90 days (3). Unfortunately, other than corticosteroids, which may convey a short-term mortality benefit (4), no other pharmacotherapy for AH is currently available, highlighting the need for new insights into the mechanisms of this deadly condition.

The role of platelets in liver pathology is an emerging area of interest. Platelets have been implicated in the progression of metabolic-associated steatohepatitis (MASH) (5), and a clinical trial demonstrated that aspirin may reduce liver fat content (6). Platelets have also been implicated in liver inflammation in COVID-19 (7) and in liver fibrosis in preclinical models of cholestatic liver disease (8). In ALD and AH, previous work has identified a potential proinflammatory role for platelets (9). Although platelets do not have a nucleus, disease-related signals can alter the transcriptome of megakaryocytes (progenitor cells of platelets in the bone marrow), and these changes can be passed along to developing platelets (10, 11). Platelet proteomics and transcriptomics have emerged as effective tools in detecting such stress induced contributions.

Platelet signaling to liver endothelial cells has been previously identified to promote inflammation. Platelets have been described to promote liver sinusoidal endothelial cell (LSEC) release of proinflammatory chemokines CCL2 and CXCL8, which can recruit T cells and neutrophils to the liver and facilitate T cell binding to liver endothelium (12). Kupffer cells, the resident macrophages of the liver, are another key cell type that bind platelets, mediating cytokine and chemokine release and leading to inflammation and fibrosis in MASH (5). In addition to direct binding, it is increasingly appreciated that, upon activation, platelets can release platelet-derived microparticles (PMPs), 100 nm to 1 μm cell fragments that can contain RNA, miRNA, and protein, that can also interact with other cells (13).

In this study, we identified transcriptomic and proteomic alterations in platelets from patients with AH. The proinflammatory changes were linked to markers of endotheliopathy, and liver dysfunction. Notably, the alarmins S100A8 and S100A9 were significantly increased in both platelets and PMPs from patients with AH, compared with healthy controls, and their levels significantly correlated with disease severity. We identified S100A9, the dominant proinflammatory signal of the 2, as a driver for LSECs to adopt a proinflammatory phenotype via crosstalk with monocytes. We additionally identified IL-6, a key inflammatory cytokine in AH (14), as a regulator of these pathological changes at the level of the megakaryocyte. These results identify what we believe is a new mechanism for platelet-mediated inflammation in AH and set the stage for what we believe would be novel platelet-targeted interventions.

## Results

### Platelets undergo proinflammatory transcriptomic changes in AH.

**Figure 1:**
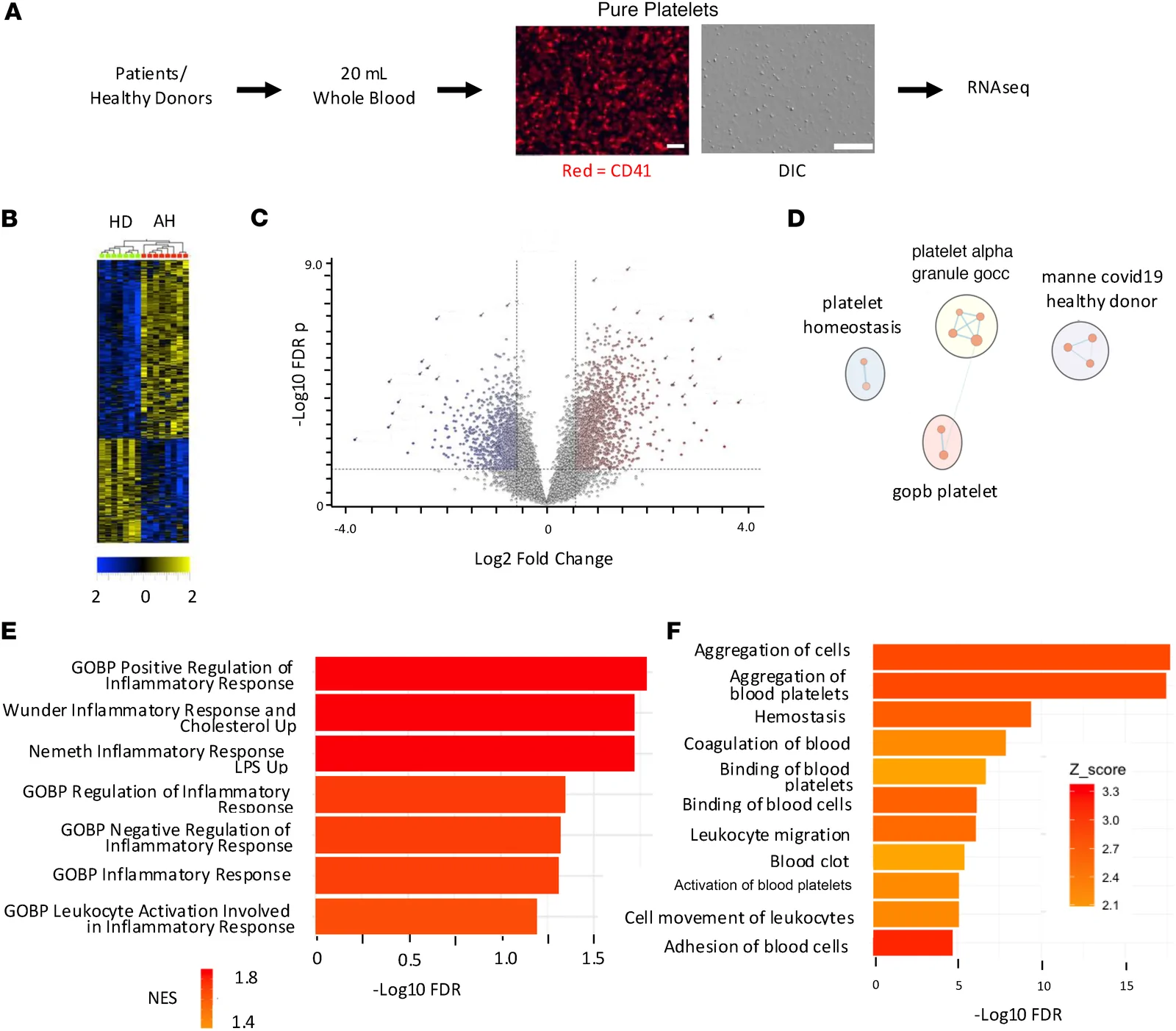
Differential gene expression and pathway analysis of platelets from patients with alcohol-associated hepatitis. (**A**) Workflow of a typical experiment for platelet RNA isolation. Red, CD41 immunofluorescence; DIC, differential interference contrast. Scale bar: 50 μm. (**B**) Heatmap of differentially expressed genes in healthy donor (HD, *n* = 7) versus alcohol-associated hepatitis (AH, *n* = 8) platelets. (**C**) Volcano plot of differentially expressed genes in platelets from HD versus AH. (**D** and **E**) Selected upregulated pathways in AH platelets versus HD platelets based on gene set enrichment analysis. (**F**) Upregulated pathways in AH platelets versus HD platelets based on Ingenuity Pathway Analysis.

We isolated pure platelets from whole blood of patients with AH (Supplemental Table 1; supplemental material available online with this article; https://doi.org/10.1172/jci.insight.201114DS1) or healthy donors (Supplemental Table 2) according to the workflow in Figure 1A. We next isolated total RNA from those platelets and performed RNA-seq to assess transcriptomic changes associated with AH pathogenesis. The DESeq2 method (15) was used to compared gene expression between the 2 groups, and genes with adjusted *P* < 0.05 and absolute log_2_ fold changes > 0.58 were considered differentially expressed genes (DEGs). Shown in the heatmap in Figure 1B and volcano plot in Figure 1C are the large array of dynamic alterations to the platelet transcriptome in AH. To understand the biological consequences of these changes, functional annotation was then performed using gene set enrichment analysis (GSEA) and results were clustered using the Enrichment Map application (16) on Cytoscape software to define overarching groups of similar gene sets. Shown in Figure 1D are clusters of platelet function pathways that reflect platelet activation (platelet α granule cluster and gene ontology biological process [GO-BP] platelet cluster), indicating that transcriptomic changes in AH platelets are reflecting activation. Disease-relevant upregulated significant gene sets (FDR *P* < 0.05) are displayed in the bar graph in Figure 1E and colored based on the value of the Normalized Enrichment Score (NES) and demonstrate the upregulation of numerous proinflammatory pathways in AH platelets. DEGs were also functionally analyzed using Ingenuity Pathway Analysis (Qiagen) and disease-relevant significant (FDR *P* of Fisher’s exact test < 0.05) pathways are shown in Figure 1F colored according to the value of the *z* score for activation. We again found evidence in these pathways of platelet activation and inflammatory processes such as leukocyte migration. Taken together, these data demonstrate heightened activation of platelets in AH and a potential inflammatory role for AH platelets.

### The platelet proteome in AH reveals upregulated proinflammatory mediators.

**Figure 2:**
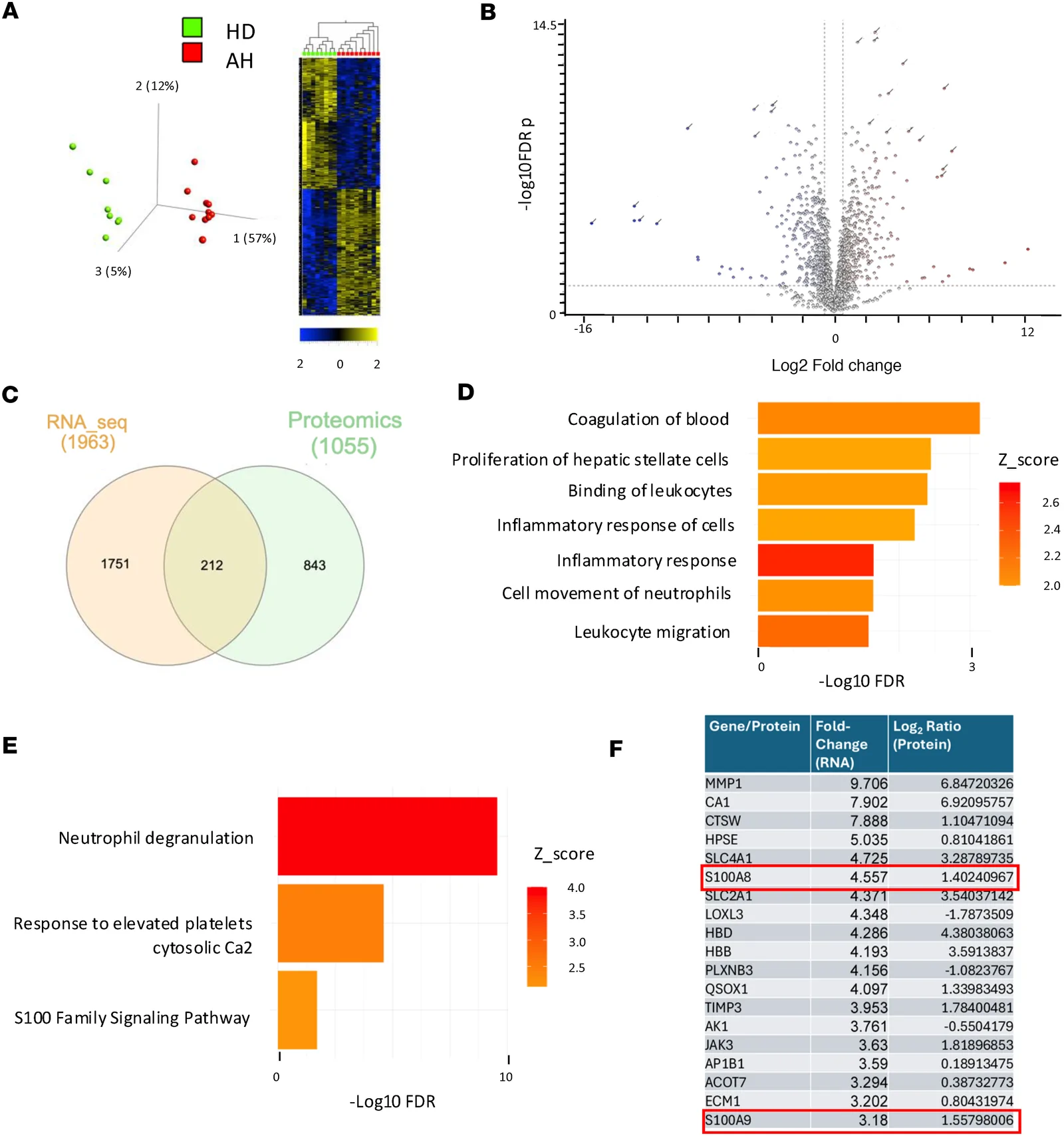
Integrated transcriptomic and proteomic analyses identify overlapping inflammatory pathways and upregulation of S100A8 and S100A9 in platelets from patients with alcohol-associated hepatitis. (**A**) Principal component analysis and heatmap of differentially expressed proteins in AH (*n* = 10) platelets versus HD (*n* = 8). (**B**) Volcano plot of differentially expressed proteins from platelets in AH versus HD. (**C**) Venn diagram showing 212 proteins and their corresponding genes that were differentially expressed and concordantly regulated in AH versus HD. (**D** and **E**) Selected ingenuity pathway analysis diseases and functions (**D**) and pathways (**E**) for concordantly regulated genes and proteins in AH platelets. (**F**) S100A8 and S100A9 were among the top 20 most upregulated genes with concordantly upregulated proteins in AH platelets by fold-change. GO-BP, gene ontology biological process; GOCC, gene ontology cellular component.

Because our data suggest a role for platelets in inflammation in AH based on gene expression, we next sought to define the platelet phenotype in AH at the protein level. Pure platelets were again isolated from patients with AH (Supplemental Table 3) and healthy donors (Supplemental Table 4) with the incorporation of an additional washing step to remove residual plasma proteins. Total protein was then extracted and analyzed with label-free quantitation proteomics. Differentially abundant proteins (DAP) were subsequently determined by 2-tailed *t* test, and proteins were termed DAPs with an adjusted *P* < 0.05. Principal component analysis (PCA) and the hierarchical clustering heatmap of DAPs are shown in Figure 2A, and a volcano plot of the global proteomic changes is shown in Figure 2B. Complementary to our transcriptomic data, these results show a large number of dynamic changes in the platelet proteome in AH, which we sought to narrow to molecules produced entirely within platelets and their precursor cells in the bone marrow, megakaryocytes. To do this, we integrated our proteomics and RNA-seq data to define molecules for whom we detected both DEGs and DAPs, shown as the overlapped 212 molecules in the Venn diagram in Figure 2C. We further narrowed to those molecules for which the fold-change of the transcript expression and protein abundance in AH versus HD was in the same direction. We used these molecules to assess significant biological functions (FDR *P* of Fisher’s exact test < 0.05) using Ingenuity Pathway Analysis, and found functions relating to blood coagulation, liver fibrosis (proliferation of hepatic stellate cells), and inflammation were represented (Figure 2D), as well as pathways relating to neutrophil degranulation, platelet activation, and the S100 family signaling pathway (Figure 2E). We next checked the top 20 genes for fold-change upregulation in AH platelets with concordant protein upregulation and found that S100A8 and S100A9, 2 proteins implicated in inflammation (17), were in these top upregulated molecules (Figure 2F). These data suggest that S100A8 and S100A9 are an important component of the platelet inflammatory signature in AH and may be playing a role in inflammation in this disease.

### Hepatic endotheliopathy and PMPs containing S100A8 and S100A9 are upregulated in AH.

**Figure 3:**
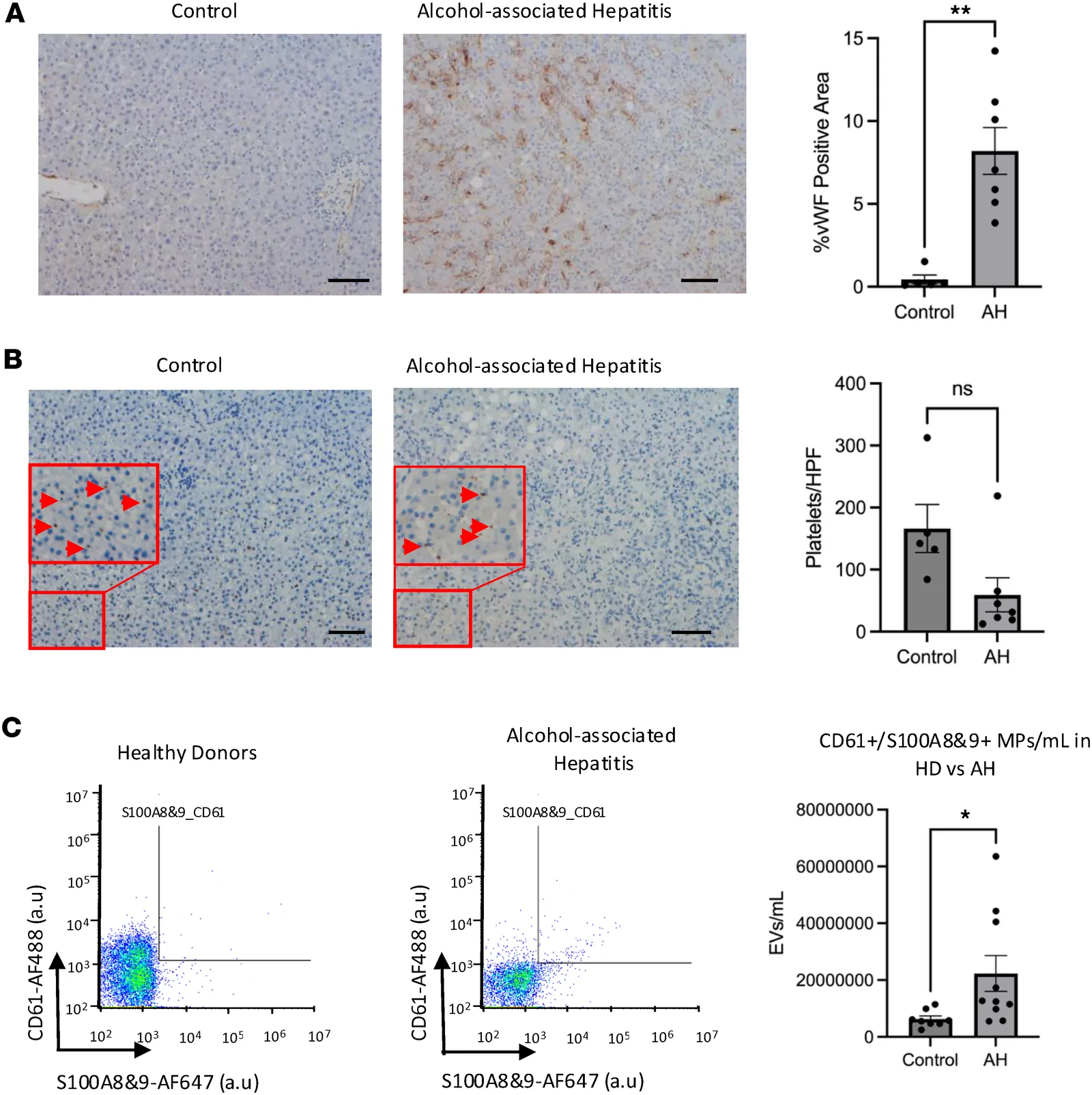
Intrahepatic platelets and circulating platelet-derived microparticles in alcohol-associated hepatitis. (**A** and **B**) IHC for von Willebrand factor (vWF) (**A**) and CD61 (**B**) in liver tissue of patients with alcohol-associated hepatitis (AH, *n* = 7) and healthy donors (HD, *n* = 5). Scale bar: 100 μm. Arrowheads point to platelets. (**C**) Nanoscale flow cytometry was performed on AH (*n* = 10) and HD (*n* = 8) plasma to quantify the number of platelet-derived microparticles double-positive for CD61 and S100A8/S100A9. Two-tailed unpaired *t* test for 2 groups with Welch’s correction was used. *P < 0.05, **P < 0.01. MP, microparticle.

Von Willebrand factor (vWF) enhances platelet adhesion to endothelial cells and, in excess, is a marker of endotheliopathy. We assessed vWF levels in the livers of patients with AH (Supplemental Table 5) or healthy donors with IHC and found significant upregulation of vWF^+^ area in AH livers (8.19% versus 0.44%, *P* = 0.001) (Figure 3A), indicating that the capacity for platelet adhesion in the liver may be enhanced. However, IHC for platelet marker CD61 revealed a trend toward fewer platelets in the liver in HD versus AH (166.2/hpf versus 59.3/hpf, *P* = 0.06) (Figure 3B), suggesting that platelets in the liver may not remain intact. Because interactions between vWF and platelet glycoprotein Ib α may shear platelets into fragments that are termed PMPs (18), we next examined these microparticles in AH. PMPs are 100 nm to 1 μm cell fragments arising from platelets upon activation, apoptosis, pyroptosis, or shear stress. In addition to vWF, highly inflammatory environments have been shown to increase platelet cell death and fragmentation (19). PMPs can carry protein, mRNA, or miRNA from platelets to other cells, including monocytes, neutrophils, and endothelial cells (20, 21) and, by some estimates, may account for 70%–90% of extracellular vesicles in the blood in healthy conditions (13). In addition, the liver has previously been demonstrated to be a major site of nanoparticle accumulation (22, 23). To investigate whether PMPs could be carrying increased proinflammatory signals such as S100A8 and S100A9 in AH, nanoscale flow cytometry was performed on plasma from the patients and healthy donors in whom platelet proteomics was previously shown. These experiments revealed that microparticles double-positive for platelet marker CD61 and S100A8/S100A9 were significantly increased in AH versus HD (2.23 × 10^7^ MPs/mL versus 6.31 × 10^6^ MPs/mL, *P* = 0.03) (Figure 3C). CD61 is a specific platelet marker, so these microparticles are specifically arising from platelets based on being CD61^+^. The data suggest that PMPs may mediate liver inflammatory signaling in AH.

### Inflammatory PMPs are positively correlated with plasma markers of endotheliopathy in patients with AH.

**Figure 4:**
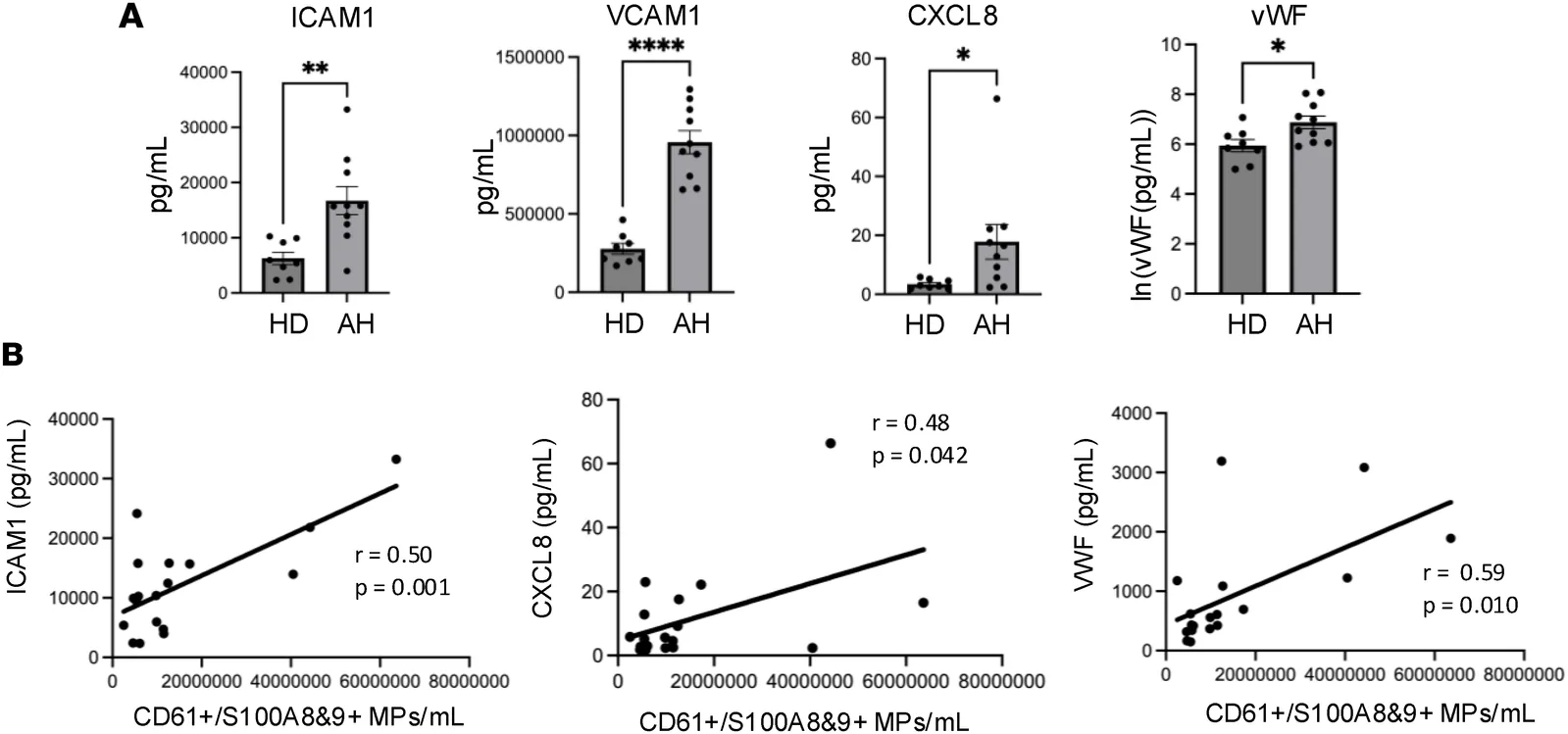
Proinflammatory markers of endotheliopathy are elevated in alcohol-associated hepatitis patient plasma and positively correlate with CD61^+^S100A8/9^+^ microparticles. (**A**) Concentrations of proinflammatory markers of endotheliopathy (pg/mL) were measured in healthy donor (HD, *n* = 8) and alcohol-associated hepatitis (AH, *n* = 10) patient plasma via Luminex assay. (**B**) CD61^+^ S100A8/9^+^ microparticles were correlated with plasma concentrations of endotheliopathy markers in patients with AH. Two-tailed unpaired *t* test for 2 groups or simple linear regression was used. *P < 0.05, **P < 0.01, ****P < 0.0001. ICAM1, intracellular adhesion molecule 1; VCAM1, vascular cell adhesion molecule 1; CXCL8, CXC motif chemokine ligand 8; vWF, von Willebrand factor; MP, microparticle.

To further study the role of PMPs in AH, we next examined their relationship with clinical indicators of disease pathogenesis and severity. Given that LSECs are the most abundant endothelial cells in the liver, play a pathological role in ALD (24–26), and are major scavengers of nanoparticles (27), we sought to define markers of endotheliopathy (a proinflammatory endothelial cell state) in relation to PMP abundance in AH. We utilized the same patient or healthy donor plasma from which we analyzed the PMP, and we performed a Luminex assay to study previously reported (28, 29) mediators of endotheliopathy or endothelial inflammation such as ICAM1, VCAM1, CXCL8, and vWF. We found that ICAM1 (62,693 versus 167,322 pg/mL, *P* = 0.003), VCAM1 (267,922 pg/mL versus 956,883 pg/mL, *P* < 0.0001) CXCL8 (3.41 pg/mL versus 17.82 pg/mL, *P* = 0.046) and ln-transformed concentration of vWF (5.942 versus 6.876 *P* = 0.02) are present at significantly higher concentrations in AH plasma versus HD (Figure 4A). To further investigate the pathological role of CD61^+^/S100A8/9^+^ microparticles, their concentration was plotted against the plasma concentrations of the inflammatory endotheliopathy markers. PMPs carrying S100A8/S100A9 were found to be significantly positively correlated with ICAM1 (*r* = 0.50, *P* = 0.001), CXCL8 (*r* = 0.48, *P* = 0.04), and vWF (*r* = 0.59, *P* = 0.01), indicating that they may have a potential mechanistic role in inflammatory endotheliopathy in AH (Figure 4B).

### S100A9 is upregulated in megakaryocytic cells via IL-6 signaling through JAK/STAT pathways.

S100A8 and S100A9 are upregulated by various cells in inflammation (17), and platelet-derived S100A8 and S100A9 have been described as promoting endotheliopathy in COVID-19 (30). S100A8 and S100A9 can be present as both heterodimers (S100A8/S100A9) or homodimers of the individual proteins. Inflammatory settings have been linked with preferential secretion of S100A9, and S100A9 homodimers are preferentially stabilized by inflammatory stimuli such as IL-1B, lipopolysaccharide (LPS), and TNF-α, which are critical to the pathogenesis of AH (31). We therefore focused on the S100A9 protein in particular as a pathological signal in AH.

**Figure 5:**
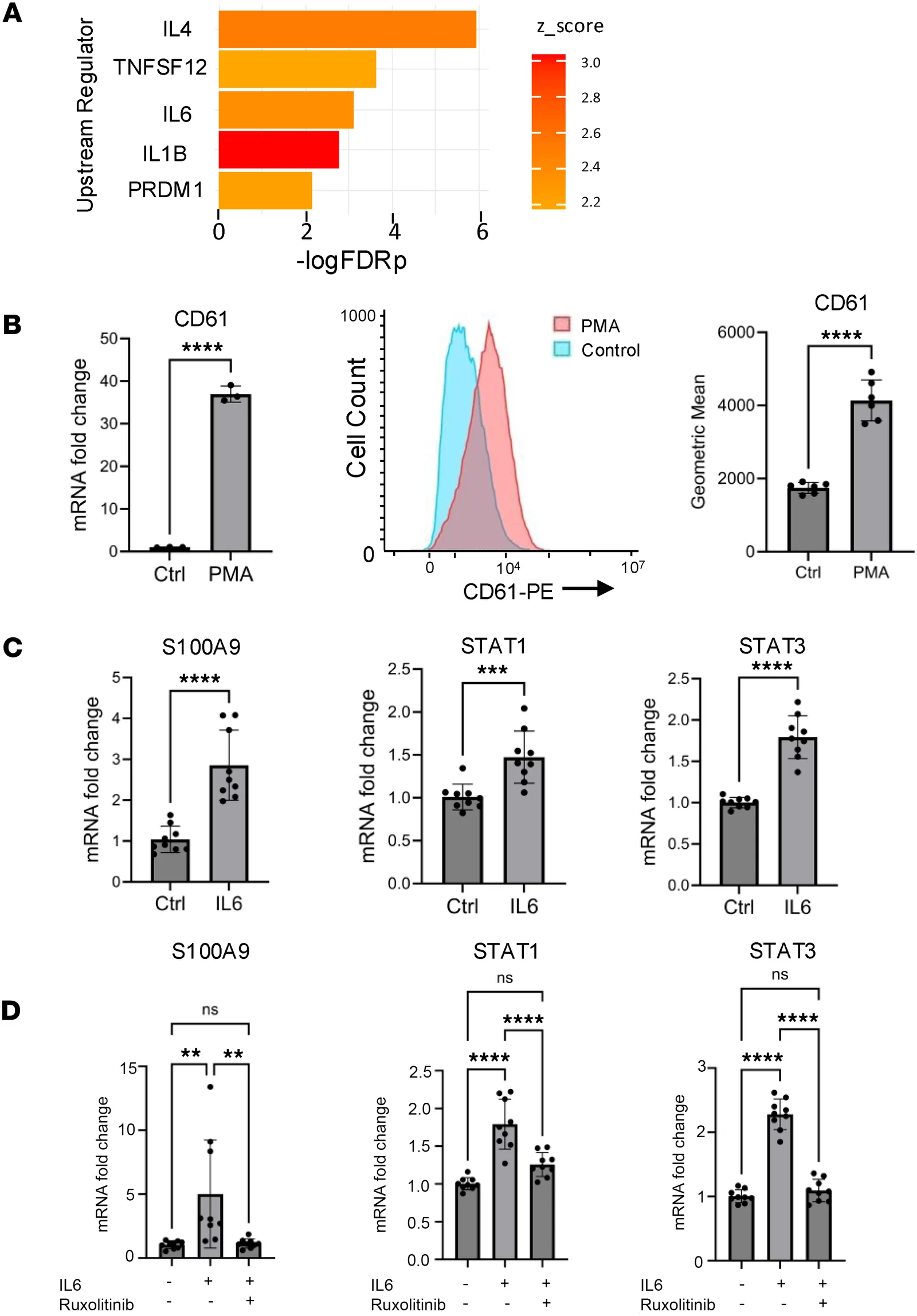
*S100A9* is upregulated via IL-6 and JAK/STAT signaling in human megakaryocytic cells. (**A**) Ingenuity Pathway Analysis was done to predict potential upstream regulators of S100A9 in our RNA-seq data from alcohol-associated hepatitis (AH) patient platelets. (**B**) qPCR for expression of CD61, a megakaryocyte marker, to assess K562 cell differentiation to a megakaryocyte phenotype with phorbol 12-myristate 13-acetate (PMA) (80 nM) compared with control. Representative image and geometric means of CD61 expression in K562 cells differentiated with PMA compared with control measured via flow cytometry. (**C**) qPCR for S100A9 and IL-6 downstream signals in K562 cells treated with PMA (80 nM) for 18 hours with or without IL-6 (20 ng/mL) for 6 hours. (**D**) qPCR for S100A9 and IL-6 downstream signals in K562 cells differentiated with PMA (80 nM) for 18 hours, pretreated with or without Ruxolitinib (2 μM) for 20 minutes followed by 6 hours, treated with or without IL-6 (20 ng/mL) for 6 hours. Data are a compilation of 3 experiments. One-way ANOVA with Tukey’s test for multiple groups, or 2-tailed unpaired *t* test for 2 groups was used. **P < 0.01, ***P < 0.001, ****P < 0.0001. CD61, integrin B3; PMA, phorbol 12-myristate 13-acetate.

Because platelets do not have a nucleus, transcriptomic changes observed in platelets are most likely to be arising at the level of their precursor cells in the bone marrow, megakaryocytes. To study the mechanism of S100A9 upregulation in platelets beginning at the mRNA level, Ingenuity Pathway Analysis was utilized for prediction of upstream regulators of the platelet transcriptomic changes we observed in AH. Possible upstream regulators with S100A9 as a potential downstream target were predicted, and given its key role in AH, we identified IL-6 as a candidate for further study (Figure 5A). Human megakaryocytes were modeled utilizing K562 cells differentiated to a megakaryocytic phenotype with phorbol 12-myristate 13-acetate (PMA) as previously described (32) and shown by upregulation of the megakaryocyte marker CD61 (Figure 5B). These differentiated cells were then treated with recombinant human IL-6 which significantly upregulated the mRNA expression of *S100A9* (2.855-fold, *P* < 0.0001), as well as *STAT1* (1.473-fold, *P* = 0.0008), and *STAT3* (1.794-fold, *P* < 0.0001) (Figure 5C). To verify the role of JAK/STAT signaling in upregulation of S100A9 via IL-6 in megakaryocytic cells, differentiated K562 cells were treated with IL-6 with or without the JAK inhibitor Ruxolitinib, which blocked IL-6–mediated upregulation of *S100A9*, *STAT1*, and *STAT3* (Figure 5D). These results indicate that platelet upregulation of S100A9 may be governed by IL-6 effects at the level of megakaryocytes.

### S100A9 promotes inflammatory endotheliopathy via crosstalk between monocytes and LSECs.

**Figure 6:**
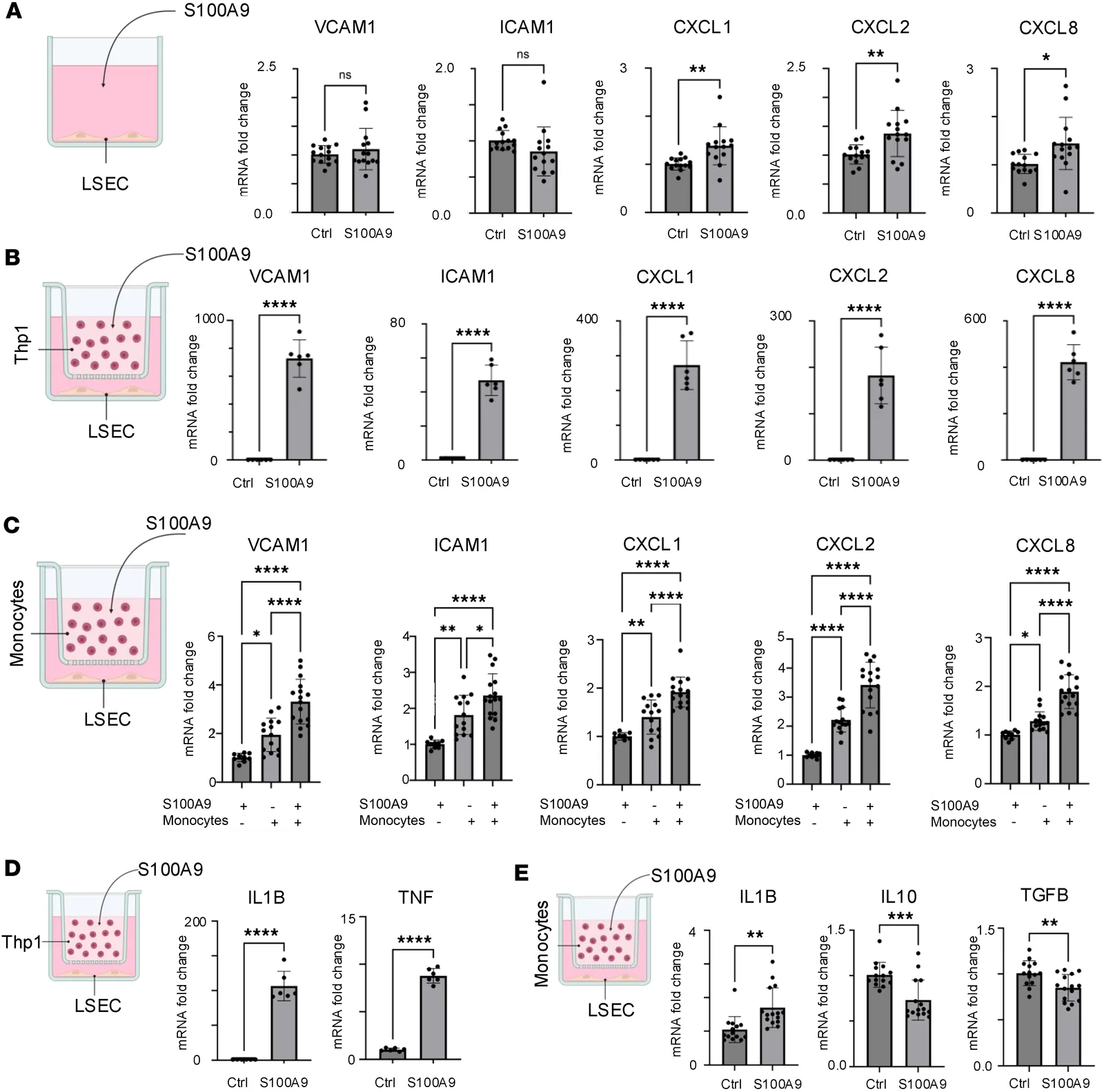
S100A9 significantly increases expression of inflammatory genes in primary human monocytes and liver sinusoidal endothelial cells. (**A**) qPCR of proinflammatory endothelial markers measured in liver sinusoidal endothelial cells (LSECs) treated with S100A9 (1 μg/mL) for 6 hours. (**B**) qPCR of proinflammatory endothelial markers measured in LSECs in coculture with Thp1 cells treated with S100A9 (1 μg/mL) for 6 hours. (**C**) qPCR of proinflammatory endothelial markers measured in LSECs in coculture with primary human monocytes treated with S100A9 (1 μg/mL) for 6 hours. (**D**) qPCR of proinflammatory markers in Thp1 cells from **B**. (**E**) qPCR of proinflammatory markers in monocytes from **C**. Graphs show the fold-change versus control. Data are a compilation of 3 experiments. One-way ANOVA with Tukey’s test for multiple groups, or 2-tailed unpaired *t* test for 2 groups was used. *P < 0.05, **P < 0.01, ***P < 0.001, ****P < 0.0001. VCAM1, vascular cell adhesion protein 1; ICAM1, intercellular adhesion molecule 1; CXCL1, CXC motif chemokine ligand 1; CXCL2, CXC motif chemokine ligand 2; CXCL8, CXC motif chemokine ligand 8. Created in BioRender.

S100A9 is known to promote inflammatory processes through signaling to cells via the TLR4 receptor and possibly the RAGE receptor (19). To investigate the inflammatory effect of S100A9 on the liver, LSECs were treated with recombinant human S100A9. The LSECs showed a pronounced inflammatory response with significant upregulation of RNA expression of inflammatory factors *CXCL1* (1.386-fold, *P* = 0.002), *CXCL2* (1.375-fold, *P* = 0.004), and *CXCL8* (1.443-fold, *P* = 0.01), but either no change or downregulation of *VCAM1* and *ICAM1*, which differed from the endotheliopathy signature found in patient plasma (Figure 6A). Because of this difference, we sought to better model the environment of the liver in AH by coculturing LSECs with monocytes, which infiltrate the liver in this disease (33) and are also active phagocytic cells for platelet microparticle clearance (34). As a first step, the human monocytic cell line Thp1 cells were cocultured with LSECs in a transwell system prohibiting direct cell-cell contact and treated with S100A9. This resulted in the significant upregulation of mRNA expression of *VCAM1* (726.7-fold, *P* < 0.0001), *ICAM1* (46.91-fold, *P* < 0.0001), *CXCL1* (272.8-fold, *P* < 0.0001), *CXCL2* (182.6-fold, *P* < 0.0001), and *CXCL8* (422.1-fold, *P* < 0.0001) in the LSECs (Figure 6B). This result was also replicated in human umbilical vein endothelial cells (HUVECs) (Supplemental Figure 1). We next repeated the coculture using primary human monocytes from healthy donors and LSECs with 2 control conditions to account for the independent effects of S100A9 and monocytes on the LSECs. It was found that *VCAM1*, *ICAM1*, *CXCL1*, *CXCL2*, and *CXCL8* were significantly upregulated in the LSECs compared with both controls (Figure 6C). These results indicate that S100A9 promotes the highest degree of inflammation and endotheliopathy through crosstalk between monocytes and LSECs as opposed to direct action on LSECs alone.

We also examined whether LSECs may upregulate CCL5, CCL2, or PDGFB, which are molecules linked with pathological signaling to hepatic stellate cells in this crosstalk system. In the Thp1 coculture system (Supplemental Figure 2A), LSECs significantly upregulated *CCL2* (16.52-fold, *P* < 0.0001), and *PDGFB* (2.05-fold, *P* < 0.0001), and in the primary human monocyte system (Supplemental Figure 2B) *CCL2* was again significantly upregulated (2.073-fold, *P* < 0.01). We also assessed whether S100A9-mediated crosstalk can alter endothelial nitric oxide synthase (*eNOS*) expression in LSECs, and found a significant decrease in *eNOS* expression in the Thp1 cell system (Supplemental Figure 2A), as well as a trend toward a decrease in the primary human monocyte system (Supplemental Figure 2B), although not reaching statistical significance. These data indicate that S100A9 may induce pathological crosstalk between LSECs and hepatic stellate cells via CCL2, and could affect LSEC functional phenotype, although the latter remains uncertain.

To begin to examine possible mechanisms of monocyte-LSEC crosstalk amplifying endotheliopathy in response to S100A9, we checked gene expression of proinflammatory factors in Thp1 cells and primary human monocytes used in our coculture system. *IL1B* (106.5-fold, *P* < 0.0001) and *TNFA* (8.786-fold, *P* < 0.0001) were significantly upregulated in the Thp1 cells (Figure 6D). *IL1B* (1.698-fold, *P* = 0.002) was significantly upregulated in the primary human monocytes, whereas *IL10* (0.7306-fold, *P* = 0.0004) and *TGFB* (0.8494-fold, *P* = 0.005) were significantly downregulated (Figure 6E), indicating that a proinflammatory monocyte phenotype and IL-1B production may be mediating the observed inflammatory endotheliopathy.

### LSEC phenotype is broadly altered in AH.

Our in vitro system was inadequate to examine changes in LSEC zonation (Supplemental Figure 2C). In order to examine changes in LSEC phenotype in AH more comprehensively, we analyzed previously reported single-cell RNA-seq data from the liver of patients with AH (35) and healthy controls (36). Cells were clustered as in Supplemental Figure 3A, and clusters annotated based on the markers in Supplemental Table 4. We identified 2 major populations of endothelial cells, those expressing high levels of canonical LSEC markers such as *STAB1*, *STAB2*, and *LYVE1* (canonical LSECs) and those expressing higher levels of more macrovascular endothelial cell markers such as *vWF*, *CD34*, and *PECAM1* (macrovascular endothelial cells) (Supplemental Figure 3B). In AH livers, we noted a pronounced loss of canonical EC identity and an increase in macrovascular endothelial cells (Supplemental Figure 3C), suggesting dedifferentiation of LSECs to a population expressing higher markers of capillarization such as CD34 and fewer markers consistent with normal LSEC function and identity. Pathway analysis in Supplemental Figure 3D showing pathways up- and downregulated in the macrovascular EC cluster in AH shows that these cells are metabolically suppressed compared with the canonical LSECs. Supplemental Figure 3E assesses typical zonation markers between macrovascular and canonical LSECs in AH and shows that the macrovascular LSECs express more pericentral and panendothelial markers. Therefore, the shift to a macrovascular EC phenotype likely reflects a loss of LSEC zonation identity in AH. Notably, the macrovascular ECs express lower levels of regenerative factor *WNT2*, suggesting they may have impaired regenerative capacity compared with canonical ECs.

### LSEC to hepatic stellate cell crosstalk in AH.

We utilized CellChat with our single-cell data to explore changes in LSEC to hepatic stellate cell signaling under healthy and diseased conditions (Supplemental Figure 3F). We note that profibrotic signals from both macrovascular endothelia and canonical LSECs are present in AH that are absent in healthy livers. These include *COL4A1*, *COL4A2*, and *LAMC1*.

### Platelet inhibition ameliorates liver pathology in an animal model of AH.

**Figure 7:**
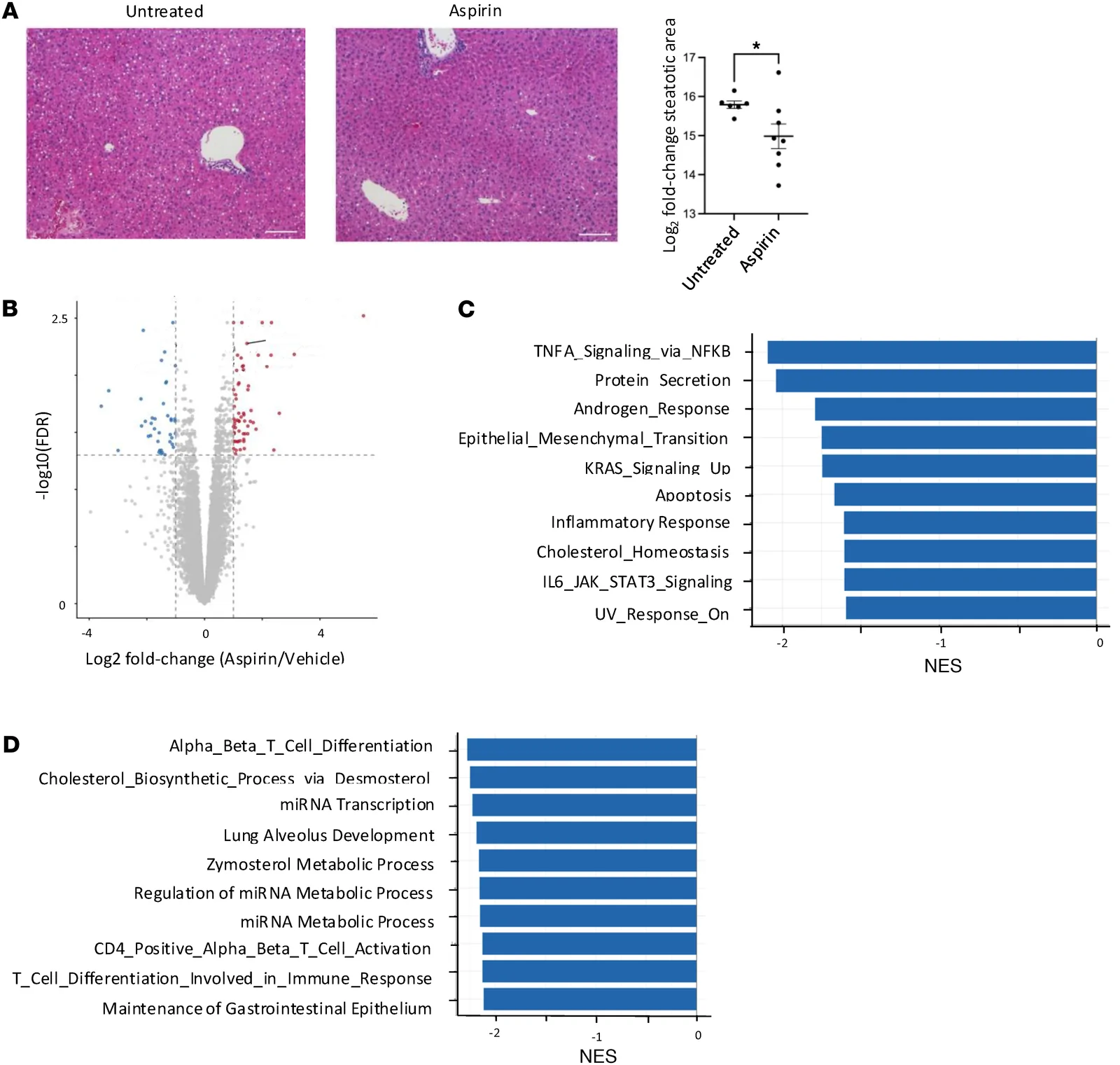
Aspirin ameliorates pathological features of alcohol-associated hepatitis. Mice treated according to the NIAAA model of AH were given daily aspirin or vehicle. (**A**) Aspirin resulted in reduced liver steatosis. Scale bar: 110 μm. Total RNA was isolated for the liver and analyzed by bulk RNA-seq. (**B**) Volcano plot of differentially expressed genes. (**C** and **D**) Hallmark pathway analysis top significantly downregulated pathways in the aspirin groups, and GO-BP pathway analysis top significantly downregulated pathways in the aspirin group. Two-tailed unpaired *t* test for 2 groups with Welch’s correction was used. *P < 0.05. GO-BP, gene ontology biological process.

To better define a role for platelets as a therapeutic target in AH, we utilized an animal model based off of the previously published NIAAA model of AH (37). All mice were fed according to a chronic-plus-binge ethanol diet, and 1 group was treated with 100 mg of aspirin via daily gavage, while a second group was gavaged with only PBS. The mice treated with aspirin showed a significant reduction in liver steatosis compared with those treated with vehicle alone (Figure 7A), indicating a potential therapeutic role for platelet inhibition in AH. In addition, total RNA from the livers of these mice was isolated and analyzed with bulk RNA-seq. A volcano plot of the DEGs between the aspirin and PBS groups is shown in Figure 7B. In order to understand the biological importance of these genes, we performed GSEA and examined the top downregulated pathways in the aspirin group by NES with FDR < 0.05 utilizing Hallmark (Figure 7C) and GO-BP (Figure 7D) pathways. We noted multiple inflammatory pathways are downregulated in the aspirin group, including TNF-α signaling, IL-6 signaling, apoptosis, and T cell–related pathways. Taken together, these data suggest a beneficial effect of platelet inhibition with aspirin on AH pathology.

### S100A9-containing platelet microparticles positively correlate with markers of AH disease severity.

**Figure 8:**
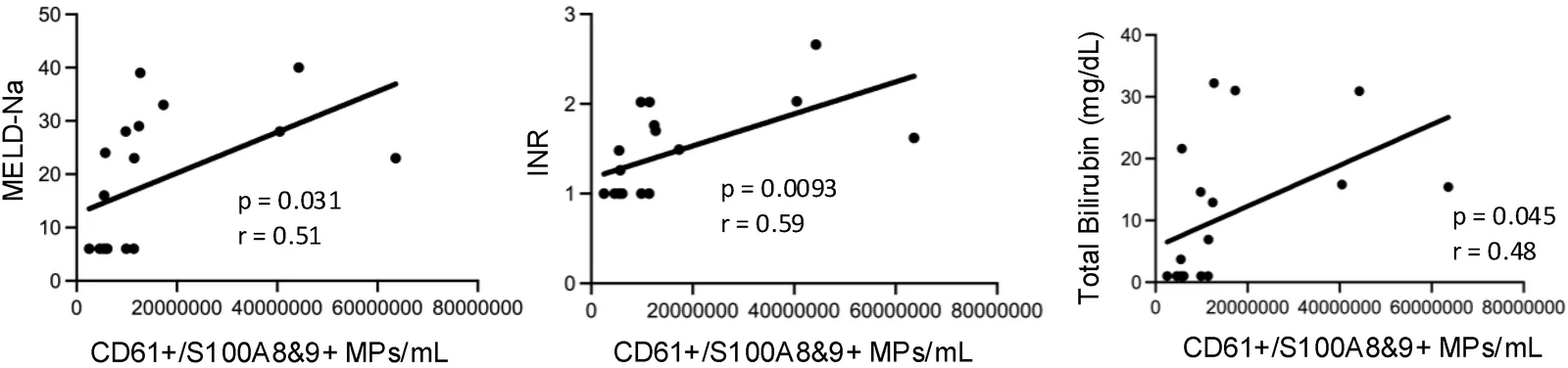
S100A9-containing platelet-derived microparticles correlate with clinical markers of disease severity in alcohol-associated hepatitis. Concentrations of CD61^+^S100A8/9^+^ PMPs have a significant positive correlation with model for end-stage liver disease sodium (MELD-Na) score (*r* = 0.51, *P* = 0.031), international normalized ratio (INR) (*r* = 0.59, *P* = 0.0093), and total bilirubin (*r* = 0.48, *P* = 0.045) in our samples (*n* = 8 healthy donors and *n* = 10 patients with AH). For healthy donors, normal values for MELD-Na (6), INR (1.0), and total bilirubin (1.0) were assumed. Simple linear regression was used.

**Figure 9:**
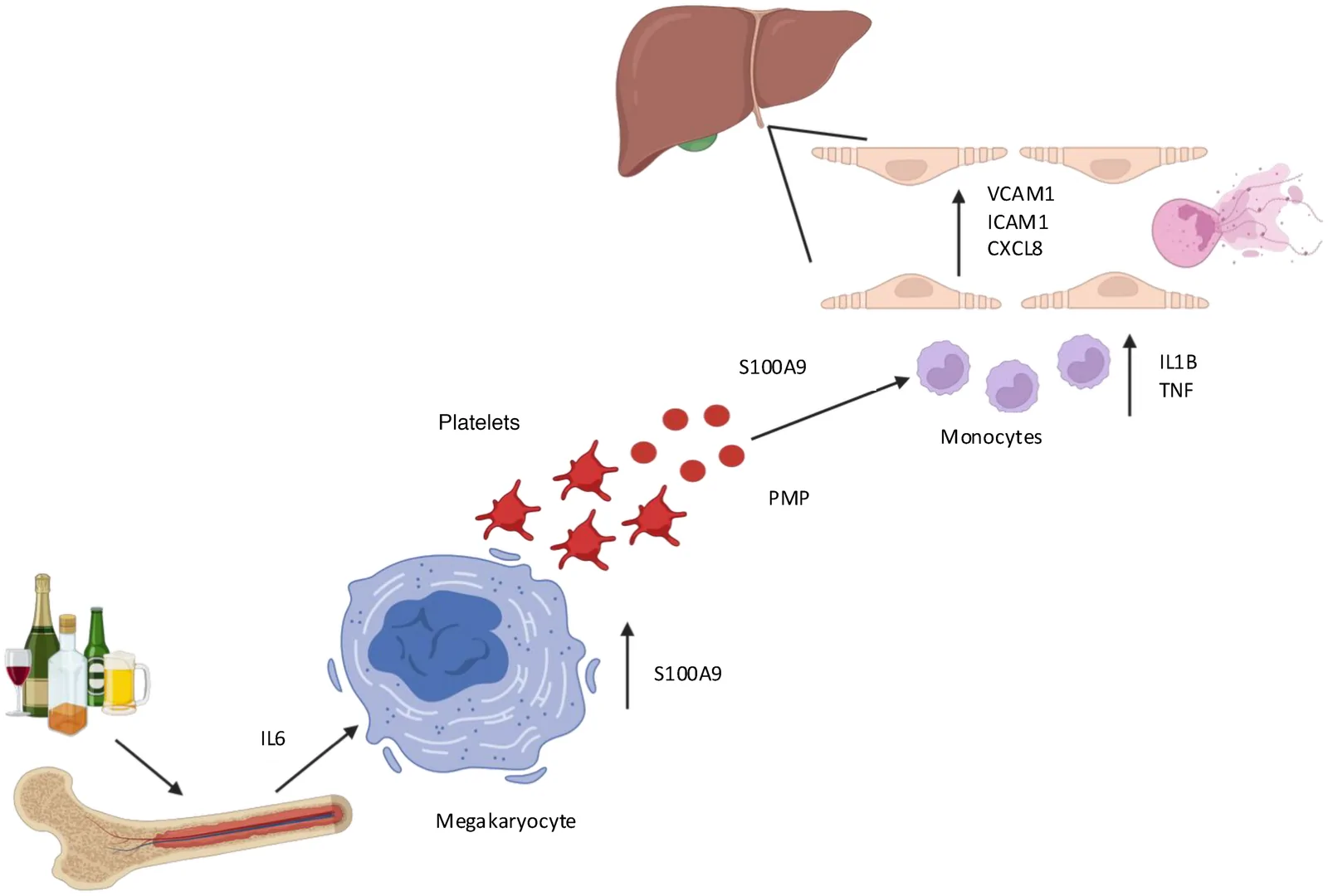
Proposed mechanism of platelet-mediated endotheliopathy and inflammation in alcohol-associated hepatitis. Our proposed mechanism by which platelets contribute to endotheliopathy and inflammation in patients. Created in BioRender.

We investigated the relationship between S100A9-containing PMPs by plotting their abundance in patients with AH and healthy donors versus key clinical parameters including model for end-stage liver disease sodium (MELD-Na) score, which denotes disease severity and prognosis in AH, along with international normalized ration (INR) and total bilirubin, laboratory tests that indicate liver dysfunction in AH (Figure 8). We found a significant positive correlation between S100A9-containing PMPs and MELD-Na score (*r* = 0.51, *P* = 0.031), INR (*P* = 0.0093, *r* = 0.59), and total bilirubin (*P* = 0.045, *r* = 0.48), suggesting a role for these PMPs in clinical severity of disease. Our proposed mechanism by which inflammatory platelets containing elevated levels of S100A9 (the more inflammatory component of the S100A8/S100A9 heterodimer) arise from the bone marrow in the context of systemic inflammation with elevated IL-6 signaling and are activated and release PMPs; these PMPs contribute to endotheliopathy, liver inflammation, and disease severity in AH is shown in Figure 9.

## Discussion

Platelets have been determined to play a pathological role in a variety of liver diseases, but their role in AH remains poorly understood. Our findings support the hypothesis that platelets undergo pathological changes in AH and promote inflammation, contributing to worsening disease. We found that platelets undergo transcriptomic and proteomic changes in AH consistent with an activated platelet phenotype, as well as the upregulation of proinflammatory pathways and proteins such as S100A8 and S100A9. A proteomic study focusing on mechanisms of platelet activation in AH in India supports our study (9). Importantly, our study focused on the United States, which has a sharply increasing number of deaths from ALD compared with other countries worldwide, and where ALD is the leading pathology for which liver transplantation is required (38). A strength of our study is our extensive use of patient samples and human cells, tissue, and plasma, which enhances the relevance of our findings to patients, especially given the limitations of recapitulating human disease in animal models of AH (39). In addition to our findings, we believe that our human transcriptomic and proteomic data will be an important shared resource for future studies of AH.

Our study explores a mechanistic role for platelets in AH by defining ways in which inflammatory platelets that have upregulated S100A8 and S100A9 may interact with the liver. Higher levels of vWF along with a trend toward lower platelet numbers in the liver in AH and the known fragmentation of platelets under inflammatory conditions (19) led us to study PMPs as a means of platelet signaling to liver cells. To our knowledge, this is the first study to target PMP in AH by nanoscale flow cytometry and demonstrate that they contain the inflammatory mediators S100A8 and S100A9. Using our clinical data, we were also able to show significant correlations between S100A8/S100A9-containing platelet microparticles and markers of endotheliopathy such as ICAM1, CXCL8, and vWF, as well as overall disease severity (MELD-Na score), suggesting a mechanistic role for PMP in both inflammatory endotheliopathy and overall patient outcomes.

Leveraging the strength of our combined transcriptomic and proteomic data, we also defined an upstream mechanism by which S100A9 can be upregulated in megakaryocytes and their descendant platelets and PMPs via IL-6. While their role as the progenitor cells for platelets has been widely studied, the role of megakaryocytes as immune and inflammatory cells is gaining increased recognition (40). Although the IL-6 receptor has been defined on megakaryocytic cells in the past (32, 40, 41), the effects of IL-6 on these cells as it relates to inflammation is not well known, apart from one study of a different model megakaryocyte cell line relating IL-6, platelet inflammatory markers, and cardiovascular risk (42). We have discovered that IL-6 can upregulate the production of inflammatory factors from megakaryocytic cells, which may contribute to inflammation in liver disease and have also shown the dependence of IL-6–mediated S100A9 production in megakaryocytic cells on JAK/STAT signaling, which has not been previously reported.

We also determined that S100A9, the dominant inflammatory protein between S100A8 and S100A9, increases the expression of proinflammatory factors in LSECs predominantly via crosstalk with monocytes. While LSECs may express TLR4, a key receptor for S100A9, the effect of the protein alone on LSECs in vitro was only modest and inconsistent. Monocytes and macrophages are known to communicate with LSECs in homeostasis and a variety of liver diseases, including ALD, during which their interactions increase inflammation (43). When either Thp1 cells or primary human monocytes and LSECs were cultured together and S100A9 was introduced, a marked inflammatory effect on LSECs was observed, possibly mediated by monocyte TNF-α or IL-1β. In particular, IL-1β has been previously shown to exert an inflammatory effect on endothelial cells in pathological conditions (44). Our data using antiplatelet therapy in an animal model of AH also suggest a mechanistic role for platelets in liver pathology and a possible therapeutic role of antiplatelet therapy. We believe our result is the first to show a role for S100A9 in LSEC-monocyte crosstalk, and provides what we think is a novel mechanism for S100A9-mediated endotheliopathy in AH.

Our study has certain limitations. It is known that other cell types, particularly monocytes and neutrophils, can produce S100A8 and S100A9 and may contribute to the pathways we have identified, although among these platelets are perhaps the most appealing therapeutic target. Furthermore, additional studies are necessary to define the detailed cell biology of how PMPs communicate with LSECs and monocytes in AH, as well as any other pathological contents of these microparticles that may exert additional effects. For maximum translational relevance, we have focused on human samples and primary human cells; however, additional studies in animal models of AH with platelet-specific gene manipulation are needed to further define the role of platelet-specific S100A9 in inflammatory endotheliopathy. With current techniques, it was not feasible to use patient-derived PMPs in cell culture experiments directly, but improving these methods will be the subject of future studies. The detailed mechanisms governing monocyte to LSEC crosstalk are another area where future investigations will be important. It is important to note that specific LSEC functional and metabolic alterations are best assessed in in vivo models, so our studies with primary human cells are limited in defining the specific effects of inflammatory platelets on these parameters. Additionally, while our single-cell analysis raises important aspects of the change in endothelial cell phenotype in AH, the detailed effects of inflammatory crosstalk promoted by AH platelets on vascular-hepatocyte cozonation and metabolic function, as well as more detailed investigations of LSEC alterations on the hepatic microenvironment utilizing single-cell transcriptomic and proteomic technologies, will need to be confirmed and further explored in future human and in vivo studies.

In conclusion, our study reveals a link between pathological changes in platelets in AH and disease severity, and a possible mechanism by which platelets promote inflammation in the liver through crosstalk between monocytes and LSECs (Figure 9). Additionally, our findings suggest that platelets could be a therapeutic target in AH.

## Methods

### Sex as a biological variable

We enrolled both male and female patients and healthy donors in our study to account for sex as a biological variable, demographics of study participants can be found in Supplemental Tables 1–3. For our animal studies, we utilized only female mice for consistency with previous studies on aspirin in steatotic liver disease (45).

### Patient selection

Hospitalized patients with AH, defined based on the clinical assessment of a hepatologist at Yale-New Haven Hospital, were included in the study. Patients were included if this diagnosis was made within 8 weeks preceding sample collection. Patients were excluded who had a diagnosis of viral hepatitis, autoimmune liver disease, hemochromatosis, Wilson disease, or α-1 antitrypsin deficiency or if metabolic-associated steatotic liver disease was felt to be the primary cause of their presentation. Patients were also excluded who had sepsis, shock, renal impairment requiring hemodialysis, active bleeding, or a diagnosed bleeding disorder other than from liver disease, or diabetes. Details of the patient cohorts for the study can be found in the Supplemental Tables 1 and 2. Donors without a known history of liver disease were recruited as healthy controls.

### Platelet isolation

Approximately 20 mL of peripheral blood was collected from healthy donors (no known history of liver disease) or patients with AH in sodium citrate tubes. Blood was then centrifuged at 280*g* for 10 minutes without brake, and the platelet-rich plasma (PRP) layer was collected leaving at least a 5 mm margin above the white blood cell layer and supplemented with 20% acid-citrate-dextrose (ACD) buffer, pH 4.5, and 10 mM EDTA (Sigma). The PRP was then centrifuged at 400*g* for 2 minutes to pellet contaminating cells, and the supernatant was centrifuged at 1,300*g* for 5 minutes or 800*g* for 20 minutes to pellet platelets. If there was concern prior to final centrifugation for contamination of other cells while removing the platelet rich plasma, platelet preparations were inspected with a hemocytometer, and if any cells larger than platelets were seen the 400*g* × 2 minutes, centrifugation was repeated until they were no longer observed. For RNA-seq, platelet pellets were lysed using Trizol (Invitrogen) and RNA was isolated per manufacturer instructions. For proteomics, platelets were resuspended in 1 mL Tyrode Buffer (pH 7.4) with 0.2% bovine serum albumin (BSA) (Sigma) freshly added and centrifuged at 3,000*g* for 5 minutes. The supernatant was then aspirated and the platelet pellet frozen at –80°C for later analysis.

### Human plasma preparation and Luminex assay

Blood was collected from patients in 10 mL EDTA tubes and centrifuged at 1,500g for 30 minutes, after which plasma was collected and stored at –80°C for further analysis. Plasma proteins were analyzed using custom Biotechne Human Luminex Discovery assays according to manufacturer instructions. Plates were read in a Luminex 200 Analyzer (Luminex) controlled by xPONENT software. Values for each analyte were determined using Belysa software (EMD Millipore) from a standard curve of log dose versus median fluorescent intensity using a 5-parameter logistic fit. All standards and samples were run in duplicate.

### Platelet bulk RNA-seq

Platelet total RNA was analyzed for integrity on the Agilent Bioanalyzer, and sequencing libraries were prepared using the Clontech Pico Input Mammalian Kit. Sequencing was subsequently performed on the Illumina NovaSeq 6000. The sequencing reads for each of the samples were aligned to the GRCh38 human reference using HISAT2 (46). Gene-level read counts were generated using StringTie2 and its prepDE.py tool (47), based on annotations from the ENCODE v27 GTF file. Sample and experiment quality metrics were generated using Picard (48), and TPM counts were generated using Ballgown (49). Sequencing data are available in the Gene Expresison Omnibus (GEO) repository (accession no. GSE333389).

### Label-free quantitation proteomics preparation

Frozen platelets were suspended in 42 μL of a 0.1% Rapigest buffer (Waters Inc.) containing 50 mM ammonium bicarbonate. Mixtures were sonicated in a 37°C water bath for 30 minutes with intermittent 1-minute vortex duration. A small aliquot was taken to estimate the amount of total protein. The protein solution was then reduced to 4 μL of 45 mM dithiothreitol (DTT, Pierce ThermoFisher Scientific, 20290) for 30 minutes at 37°C and then allowed to cool to room temperature. In total, 4 μL of a 100 mM iodoacetamide (Sigma-Aldrich, I1149) was used to alkylate the cysteine in the dark at room temperature for 30 minutes. In-house standard methanol/water/chloroform protein precipitation like that performed in Wang et al. (50) was then conducted. The collected protein pellets were airdried and stored at –80°C until further work. Protein pellets were then dissolved in 50 μL of 8M urea containing 400 mM ABC (ammonium bicarbonate), and nanodrop used to calculate protein amount. In total, 100 μg of protein from each sample was transferred into a new tube and diluted up to 50 μL with 8M urea in 400 mM ammonium bicarbonate. Further dilution (e.g., addition of 146 μL water) was conducted prior to adding 4 μL of a stock 0.5 μg/μL trypsin for enzymatic digestion overnight at 37°C.

Digestion mixtures were quenched (with 10 μL of 20% TFA) and stored at –20°C until a desalting step with BioPureSPN PROTO 300 C18 Macro spin columns (The Nest Group, HMM S18V). The effluents from the desalting step were dried and redissolved in 50 μL of buffer solution (LB, 98% H_2_O, 2% ACN, and 0.2% TFA). An aliquot was taken, concentration measured via Nanodrop, and diluted to 0.06 μg/μL with LB. A 1:4 dilution of 10X Pierce Retention Time Calibration Mixture (catalog 88321; RTCalMix) was added to each sample prior to injecting on the UPLC Q-Exactive HFX mass spectrometer for LFQ data collection for system QC/normalization if necessary. In total, 250 ng peptides were injected into the LC-MS/MS system.

### Label-free quantitation proteomics data collection

LC-MS/MS was performed using a Waters ACQUITY UPLC M-Class system (Waters Corporation) connected to a Q-Exactive HFX (ThermoFisher Scientific) mass spectrometer. Samples were loaded into a trapping column (nanoEase M/Z Symmetry C18 Trap column, 180 μm × 20 mm) at a flow rate of 10 μL/min and washed for 3 minutes. Peptide separation was conducted with a C18 column (nanoEase M/Z column Peptide BEH C18, 75 μm × 250 mm). The compositions of mobile phases A and B were 0.1% formic acid in water and 0.1% formic acid in acetonitrile, respectively. The peptides were separated and eluted at 300 nL/min. Total run time was 225 minutes. The gradient started with 3% B at initial conditions, 6% B at 2 minutes; 25% B at 175 minutes; 40% B at 195 minutes; and 90% B from 200 to 210 minutes and back to initial 3% B condition at 212 minutes. The column was equilibrated for 13 minutes prior to the next injection. MS was acquired in profile mode over the 350–1,500 *m/z* range, 120,000 resolution, AGC target of 3E6, and a maximum injection time of 50 ms. Data-dependent MS/MS were acquired in centroid mode on the top 20 precursors per MS scan, 30,000 resolution, AGC target of 1E5, maximum injection time of 50 msec, and an isolation window of 1.4 *m/z*. Precursors were fragmented by HCD activation with a normalized collision energy of 30%. MS/MS were collected with a minimum AGC target of 1E4, and dynamic exclusion was set to was set to 30 seconds.

### Differential gene expression

The gene counts matrix was used for downstream differential expression analysis. DESeq2 method (15) was used to compare gene expression between the 2 groups (AH versus HD). Normalized counts from 2 different RNA-seq analyses were merged and corrected for batch effect using the limma package (51) on Partek Flow (Partek, Illumina, version 12.7.0). Genes with adjusted *P* < 0.05, and absolute log_2_ fold changes > 0.58 were considered DEGs. Qlucore Omics Explorer version 3.9 (Qlucore) was used for visualization including PCA, hierarchical clustering heatmap, and the global transcriptional change across the groups by a volcano plot.

### Proteomics data analysis

Collected LC-MS/MS label free quantitative (LFQ) mass spectral data were analyzed utilizing Progenesis QI software (v.4.2, Waters Inc.). Mass spectral features extraction, chromatographic/spectral alignment, data filtering, and statistical analysis were performed with the Progenesis QI software. The features were tagged in sets based on characteristics such as the number of MS/MS > 1, and *P* < 0.05. The MS and MS/MS collected for the experiment were filtered to exclude spectra with rank > 10 or isotope > 3 to ensure that the highest quality MS/MS spectral data are utilized for peptide assignments and subsequent protein ID. The remaining MS/MS and the MS features were exported to an “.mgf” (Mascot generic file) for database searching using an in-house Mascot Search Engine Search (52) which was carried out against the Swiss Protein database with taxonomy restricted to *Homo Sapiens*. Carbamidomethyl (Cys), oxidation of Met, Phospho (Ser, Thr, Tyr), Deamidation (Asn, Asp), Acetyl (Lys), and Acetyl (Protein N-Term) were entered as variable modifications. Two missed tryptic cleavages were allowed, precursor mass tolerance was set to 10 ppm, and fragment mass tolerance was set to 0.02 Da. The significance threshold was set based on a FDR of 1%. An .xml file of the Mascot search result was created before being imported into the Progenesis QI software, where search hits (peptides ID) were assigned to corresponding features, and protein quantitation were then calculated by the Progenesis QI software utilizing the sum of all unique normalized nonconflicting (peptide ID only found in one protein) peptide ions for a specific protein on each run. Results were entered into an Excel Spreadsheet for downstream biostatistics and bioinformatics. The MS proteomics data have been deposited to the ProteomeXchange Consortium via the PRIDE (53) partner repository with the dataset identifier PXD078511.

### Differential analysis of proteomics data

Normalized protein abundance was used for differential analysis. DAPs between the groups were determined by 2-tailed *t* test. Resulting proteins with adjusted *P* < 0.05 differentially were considered DAPs. Qlucore Omics Explorer version 3.9 (Qlucore) was used for visualization including PCA, hierarchical clustering heatmap, and the global proteomics change across the groups by a volcano plot.

### Functional analysis of transcriptome and proteome

DESeq2 normalized counts or normalized abundant proteins were loaded to GSEA (4.3.2, build 13, UCSD). This method determines whether a defined set of genes (e.g., pathway, biological functions) shows statistically significant, concordant differences between 2 biological states. Molecular Signature database (MSigDB v2025.1.Hs) collections used were H, hallmark gene sets; C2, pathways; C5, Gene Ontology all; and C7, ImmuneSigDB subset. Resulting enriched gene sets from GSEA (FDR *P* < 0.25) were further clustered using Enrichment Map application (version 3.5.0) (16) on Cytoscape software (version 3.7) (54) to find overarching functional annotations resulting from similarities between gene sets. In addition, DEGs, and DAPs were uploaded to Ingenuity Pathway Analysis (Qiagen, version 145030503) to determine enriched pathways, upstream regulators, and biological functions.

### Interaction network analysis of transcriptomics and proteomics

STRING knowledgebase version 12.0 (55) was used to analyze the interaction network (confidence score ≥ 0.4, FDR stringency 5%) between DEGs and DAPs. To extract clusters of interaction between proteins and transcripts, clustering analysis was done using Markov Cluster Algorithm (56) with inflation parameter 3. Cytoscape software (version: 3.10.3) was for visualization of interaction network and to r to overlay expression data for each gene (57). Central genes (aka hubs) in the network were identified by Betweenness using Cytoscape app Cytohubba (58).

### Comparison between transcriptomics and proteomics

The consistency of proteomics and transcriptomics changes was compared. Genes and proteins with FDR *P* < 0.05 for disease association on both transcript expression levels and protein abundance were selected. Log_2_ fold changes were compared for each gene. Those genes with consistent changes were further analyzed using Ingenuity Pathway Analysis software (Qiagen, version 145030503) to determine enriched pathways, upstream regulators, and biological functions (Fisher’s exact test FDR *P* < 0.05). The interaction between upstream regulators, DEGs, and relevant functions was analyzed by using IPA My Pathways tools. Graphic of results were generated using RStudio (Posit Software, version 2025.05.0+496).

### IHC

Liver tissue from patients with severe AH requiring liver transplantation or healthy donors was obtained from the NIAAA repository at Johns Hopkins University. Clinical data from healthy donors are not available, but clinical data for patients with AH can be found in the Supplemental Data. IHC for CD61 (Leica, PA0308) was performed by the Yale-New Haven Hospital clinical laboratory. IHC for vWF was performed by Yale Research Histology (vWF antibody code A0082; dilution 1:4,000; Dako). Slides were visualized on an Olympus BX51 microscope and quantification was performed with Fiji software.

### Immunofluorescence

Purified platelets from a healthy donor were allowed to settle on a glass coverslip, fixed with paraformaldehyde, and then stained with anti-CD41 (Santa Cruz, sc-365938) primary antibody followed by Alex-647 donkey anti-mouse secondary antibody (Thermo Fisher Scientific, A31571). Cells were then visualized on a Zeiss Axio Observer microscope.

### Extracellular vesicle labeling and nanoscale flow cytometry

Antibodies against CD61 (VI-PL2, 336402, BioLegend) and Calgranulin (27E10, sc-33714, Santa Cruz Biotechnology) were conjugated with Alexa Fluor 488 and 647, respectively (A88062, A88068, Thermo Fisher Scientific). Plasma samples were thawed at 37°C for 5–10 minutes and centrifuged at 13,000*g* for 5 minutes to remove aggregates. Ten μL of plasma diluted 1:20 in Dulbecco’s Phosphate-Buffered Saline (DPBS) were mixed with 10 μL of fluorescently labeled anti-CD61 and anti-Calgranulin for 30 minutes at room temperature and in the dark. DPBS was added to stop the reaction at a final volume of 200 μL. High-resolution nanoscale flow cytometry (Apogee A60-MicroPlus, Apogee Flow Systems) was used to quantify EV concentrations as previously described (59). Each sample was run in triplicate and label-free plasma samples were used as negative controls. Flow cytometry data were analyzed with FlowJo Software to determine and apply gates and generate reports with scatter plots and EV concentrations for each combination of markers and data summaries. Data acquisition and analysis was performed blinded to patient information and clinical data.

### Cell culture

HUVEC were obtained from the Yale Vascular Biology and Therapeutics Program. HUVEC were cultured in PromoCell Endothelial Cell Growth Medium MV2 (catalog C-22022) with 5% fetal calf serum (PromoCell), media supplement recommended by the manufacturer (5 ng/mL recombinant human epidermal growth factor, 10 ng/mL recombinant human basic fibroblast growth factor, 20 ng/mL recombinant human insulin-like growth factor [Long R3 IGF], 0.5 ng/mL recombinant human vascular endothelial growth factor 165, 1 μg/mL ascorbic acid, 0.2 μg/mL hydrocortisone [PromoCell]), and 1% penicillin/streptomycin (Gibco). Media were changed every 48 hours or when passaged. HUVEC were not used beyond Passage Number 7. In all experiments, HUVEC were plated in a 12-well plate coated with fibronectin (EMD Millipore Corp) at a density of 3.5 × 10^4^ cells per well in 1 mL of media.

#### LSECs.

Primary human LSECs were purchased from PeloBiotech (PB-CH-153-5511; PeloBiotech). LSECs were cultured in complete PromoCell Endothelial Cell Growth Medium MV2 (catalog C-22022) as for HUVECs. Media were changed every 48 hours or when passaged. LSECs were not used beyond Passage Number 7. LSEC were plated in a 24-well plate coated with fibronectin at a density of 3.5 × 10^4^ cells per well in 1 mL of media. The cells were directly treated with 1 μg/mL of recombinant S100A9 protein (R&D Systems, 9254-S9-050) for 6 hours.

#### K-562 cells.

K562 Cells were purchased from American Type Culture Collection (ATCC, CCL-243). K562 were cultured in Iscove’s Modified Dulbecco’s Medium (IMDM) (ATTC) with 10% heat-inactivated-FBS (Sigma), and 1% penicillin/streptomycin. Media were changed every 48 hours or when passaged. K562 cells were passaged to a density of 2.5 × 10^5^ cells/mL. K562 cells were plated in a 6-well plate at a density of 3 × 10^5^ cells per well in 1 mL of media and differentiated using 80 nM PMA (Sigma, P1585) for 18 hours. In some experiments, cells were pre-treated with 2 mM Ruxolitinib (Tocris, 7064) for 20 minutes. They were subsequently treated with varying combinations of 80 nM PMA, 20 ng/mL recombinant human IL-6 protein (R&D Systems, 206IL010) and 2 μM Ruxolitinib (Tocris, 7064) for 6 hours via a media change. Upon completion of the differentiation procedure, cells were extracellularly stained using PE-CD61 (Invitrogen, 12-0619-42) and run on a Cytoflex LX (Beckman Coulter) flow cytometer to measure CD61 expression.

#### Thp1 cells.

Thp1 were purchased from ATCC (catalog TIB-202) and cultured in RPMI medium (Gibco), 10% nonheat inactivated FBS (Sigma), and 1% penicillin/streptomycin. In total, 2 mL of media were added every 48 hours, and complete media changes were performed every 7 days or when passaged. Thp1 were passaged to a density of 4 × 10^5^ cells/mL. Thp1 were used for experiments once they had achieved a density of 1 × 10^6^ cells/mL or greater in a T75 flask. Thp1 were plated in 24-well dishes at a density of 2.5 × 10^5^ cells per well in 500 mL and starved for 24 hours in 0.1% serum media prior to use in coculture.

#### Primary human monocytes.

Whole blood was collected from a healthy donor and transferred to a SepMate tube (Stemcell Technologies, 85450). Peripheral blood mononuclear cells were then isolated per manufacturer instructions. Subsequently, monocytes were positively selected via magnetic associated cell sorting using CD14 microbeads (Miltenyi Biotec, 130-050-201) according to the manufacturer protocol. The cells were plated in a HTS transwell-24 well plate 0.4 μm polycarbonate membrane insert (Corning, 3396) at a density of 5 × 10^5^ cells/mL and serum-starved for 1 hour. Once isolated, the cells were cultured for 72 hours before use in an experiment.

#### Coculture of LSECs and primary human monocytes.

The complete endothelial cell media were aspirated from the wells containing LSECs and replaced with 500 μL of RPMI with10% Hi-FBS and 1% penicillin/streptomycin.

The media were aspirated from each well of the HTS transwell 24-well plate with 0.4 μm polycarbonate membrane insert (catalog 3396) containing the primary human monocytes. The insert was moved to the 24-well plate with the LSECs. RPMI with10% Hi-FBS and 1% penicillin/streptomycin was added to each insert and the cells were treated with 1 μg/mL of recombinant S100A9 protein (R&D Systems, 9254-S9-050) for 6 hours.

#### Coculture of HUVECs or LSECs with Thp1.

The complete endothelial cell media were aspirated and replaced with 500 μL of RPMI with 10% Hi-FBS and 1% penicillin/streptomycin. A HTS transwell 24-well plate with 0.4 μm polycarbonate membrane insert (catalog 3396) was placed on top of the LSECs (or HUVECs). The Thp1 cells were transferred from the 24-well plate to the insert and treated with 1 μg/mL of recombinant S100A9 protein (R&D Systems, 9254-S9-050) for 6 hours.

### Animal experiments

Female mice (C57BL/6J, 000664, The Jackson Laboratory) at 12–13 weeks of age were acclimatized to a liquid diet for 5 days, before being fed similarly to the NIAAA chronic-plus-binge ethanol feeding protocol. Mice were fed a 5% Lieber-DeCarli ethanol diet for 10 days, followed by a single 31.5% ethanol 5 g/kg binge gavage on the final day. Mice were treated by gavage daily with either PBS or 100 μg of acetylsalicylic acid (aspirin) dissolved in PBS from the start of the ethanol feeding. Collection of liver tissue was done approximately 9 hours after the binge.

### RNA isolation

Cells were lysed using a mixture of 10 μL 2-mercaptoethanol per every 1,000 μL RLT Plus Lysis Buffer from the RNeasy Plus Micro Kit (Qiagen, 74034). RNA was isolated from the cell lysates using the QIAcube Connect Device (9002864; Qiagen) per manufacturer instructions.

In total, 5–10 mg of frozen mouse liver tissue were lysed using a mixture of 10 μL 2-mercaptoethanol per every 1,000 μL RLT Plus Lysis Buffer from the RNeasy Plus Mini Kit (Qiagen, 74134) and a tissue homogenizer. The samples were centrifuged at 12,000*g* for 3 minutes. Supernatant was removed and transferred to a lysis tube and loaded into the QIAcube Connect Device (9002864; Qiagen). RNA was isolated from the tissue lysate per manufacturer instructions.

### RT-PCR

RNA concentration was measured using the Thermo Scientific NanoDrop 2000c spectrophotometer. cDNA was synthesized using the iScript cDNA Synthesis Kit (1708891; Bio-Rad) and the BioRad T100 Thermal Cycler. RT-PCR was subsequently performed using Taqman Primers (Thermo Fisher Scientific; full list in Supplemental Table 5) on a QuantStudio 6 Flex instrument (Thermo Fisher Scientific). GAPDH was used as the housekeeping gene.

### Single-cell RNA-seq analysis

Raw gene expression matrices were loaded into Seurat (60) for 5 AH (GSE143318) and 6 healthy (GSE136103) liver samples. Only genes expressed in a minimum of 3 cells and cells expressing a minimum of 200 genes were retained. Quality control was done by filtering cells using the following thresholds: minimum of 200 and maximum of 8,000 unique genes detected per cell, a minimum of 500 and a maximum of 100,000 total UMI counters per cell, less then 20% mitochondrial gene expression, and less than 60% ribosomal gene expression. Doublets were detected and removed using scDblFinder (61). Each sample was normalized individually using SCTransform (via glmGamPoi for efficiency) (62), and highly variable genes were identified across samples, using the top 3,000 variable genes for dimensionality reduction. All 11 samples were merged into a single Seurat object, and principle component analysis was performed across 50 components. Batch correction was performed using Harmony (63), and the first 30 Harmony-corrected principal components were used for all downstream analyses. A shared nearest-neighbor (SNN) graph was constructed using the 30 Harmony-corrected dimensions, and unsupervised clustering was performed using the Leiden algorithm as part of the Seurat FindClusters function at a resolution of 0.3. Cluster marker genes were identified using FindAllMarkers, retaining only positively enriched genes with a minimum log_2_ fold-change of 0.25 and expressed in at least 25% of cells within a cluster (adjusted *P* < 0.05). SCT model parameters were prepared prior to marker testing using PrepSCTFindMarkers. Cells were annotated using markers as in Supplemental Table 4. Two prominent clusters of liver endothelial cells were noted, and based on assessing the top upregulated genes in each cluster, they were annotated as “Macrovascular ECs” and “Canonical ECs.” The propeller function from the speckle R package (64) was used on the 3 subsets of endothelial cells (canonical LSECs, lymphatic ECs, and macrovascular ECs) to test for differences in endothelial cell type proportions between AH and healthy conditions, with the Benjamini-Hochberg method used for correction for multiple testing. Further pathway analysis of macrovascular ECs was done using clusterProfiler and Gene Ontology enrichment analysis using enrichGO. For defining zonation markers expressed in macrovascular ECs versus canonical LSECs, a curated marker of zonation-associated genes that were statistically significant between the 2 clusters after correcting for multiple comparisons were visualized in an expression matrix grouped by zone and colored by the *z*-score of the scaled average expression. To analyze cell-cell communication between liver endothelial cells and hepatic stellate cells, we utilized CellChat (65) and communication probabilities were computed using the trimean method. Cell type pairs with fewer than 10 cells in either population were excluded, and pathway-level communication probabilities were calculated by aggregating individual interaction probabilities using computeCommunProbPathway, and overall communication networks were summarized using aggregateNet. Interactions with *P* < 0.05 are shown.

### Bulk RNA-seq of mouse liver tissue

Total RNA isolated from the livers of aspirin and PBS-treated mice was sent to Plasmidsaurus for sequencing using an Illumina NovaSeq X Plus (catalog 20084804).

RNA-seq read-count matrices from 2 sequencing batches were imported in R. Count columns were renamed to base sample identifiers, and missing values after merging were set to zero. The 2 batches were merged by gene_id and gene_name, and an integer gene-by-sample count matrix was constructed.

Differential expression was performed with edgeR. Lowly expressed genes were filtered using filterByExpr, library sizes were normalized with TMM (calcNormFactors), and a generalized linear model was fit using quasi-likelihood methods with a design including batch as a nuisance covariate and condition as the variable of interest (~ batch + condition). Aspirin versus PBS effects were tested using glmQLFTest on the condition coefficient, and *P* values were adjusted for multiple testing using the Benjamini-Hochberg method (FDR).

GSEA was performed using fgsea with MSigDB Hallmark and GO:BP gene sets for *Mus musculus* obtained via msigdbr. Genes were ranked by a signed statistic defined as sign(log_2_FC) × −log_10_(*P*Value), collapsed to 1 value per gene symbol by taking the maximum statistic for duplicated symbols, and enrichment was computed with 10,000 permutations. Sequencing data are available in the Gene Expression Omnibus repository (accession no. GSE333391).

### Statistics

Bioinformatic analysis of RNA-seq data and proteomics data was performed as described above. Data are shown as the mean ± SEM unless otherwise stated. Clinical data in Supplemental Tables 1–3 are shown as median (IQR). Statistical significance was determined by performing Student’s or Welch’s *t* test, ANOVA, or linear regression as appropriate, with *P* < 0.05 considered statistically significant.

### Study approval

Human studies were approved by the Yale Human Research Protection Program Human Investigation Committee (HIC 1005006865 and HIC 0603001208). All studies were performed in accordance with the Declaration of Helsinki.

### Data availability

Primary data underlying figures are available in the Supporting Data Values file. RNA-seq and proteomics data are deposited in the repositories noted above. Any clarifications or additional requests may be addressed to the corresponding author.

## Author contributions

FK designed and conducted experiments, analyzed data, and wrote the manuscript. NF designed and conducted experiments and analyzed data. RGM analyzed data and wrote the manuscript. FC, WW, and TTL collected data, analyzed data, and wrote the manuscript. YK and FL collected data, analyzed data, and wrote the manuscript. ZS collected and provided human liver tissue. JH critically reviewed data and wrote the manuscript. YI critically reviewed data and wrote the manuscript. MJM designed experiments, conducted experiments, analyzed data, and wrote the manuscript.

## Conflict of interest

The authors have declared that no conflict of interest exists.

## Funding support

This work is the result of NIH funding, in whole or in part, and is subject to the NIH Public Access Policy. Through acceptance of this federal funding, the NIH has been given a right to make the work publicly available in PubMed Central.

- 5K08AA029182 to MJM.
- Yale Liver Center award NIH P30DK034989.
- 5R24AA025017 to ZS.
- NIH Award 1S10OD030363-01A1 (Yale Center for Genome Analysis).
- NIH Awards S10OD02365101A1, S10OD019967, and S10OD018034 (MS & Proteomics Resource Center at Yale University).

## Supplementary Material

Supplemental data

Supporting data values

## Version 1 08/24/2026

Electronic publication
