# Supplementary material for: Platelet-derived S100A9 contributes to endotheliopathy in alcohol-associated hepatitis
Source: JCI Insight. 2026 Aug 24;11(16):e201114. doi: 10.1172/jci.insight.201114 (PMC13502174; doi:10.1172/jci.insight.201114)
Supplement: Supplementary file 1 [file jciinsight-11-201114-s208.pdf]

Supplementary Figure 1

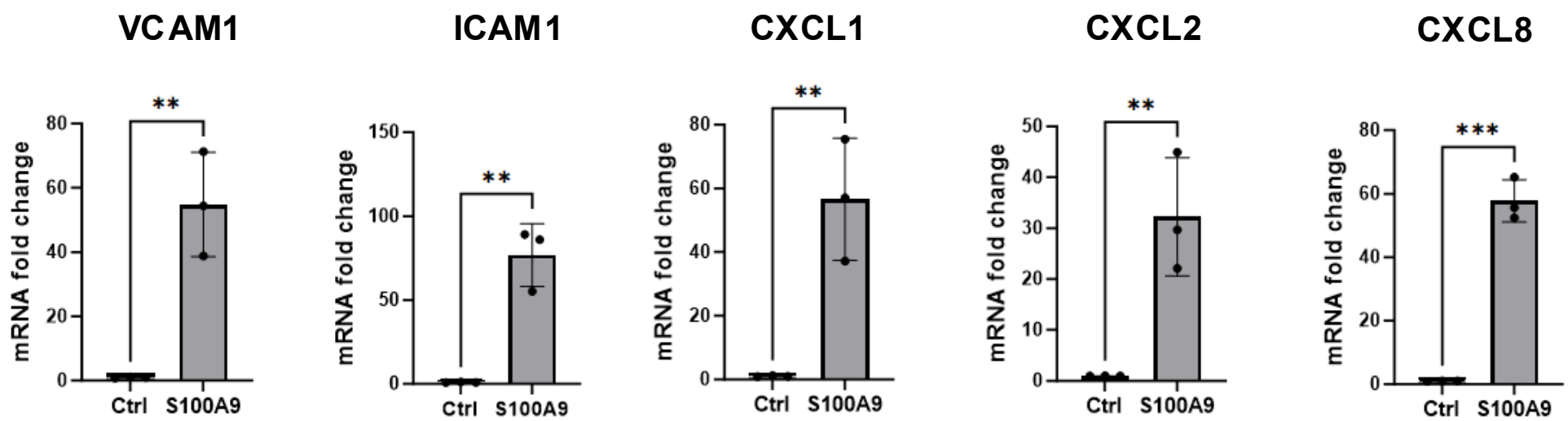

Supplementary Figure 2

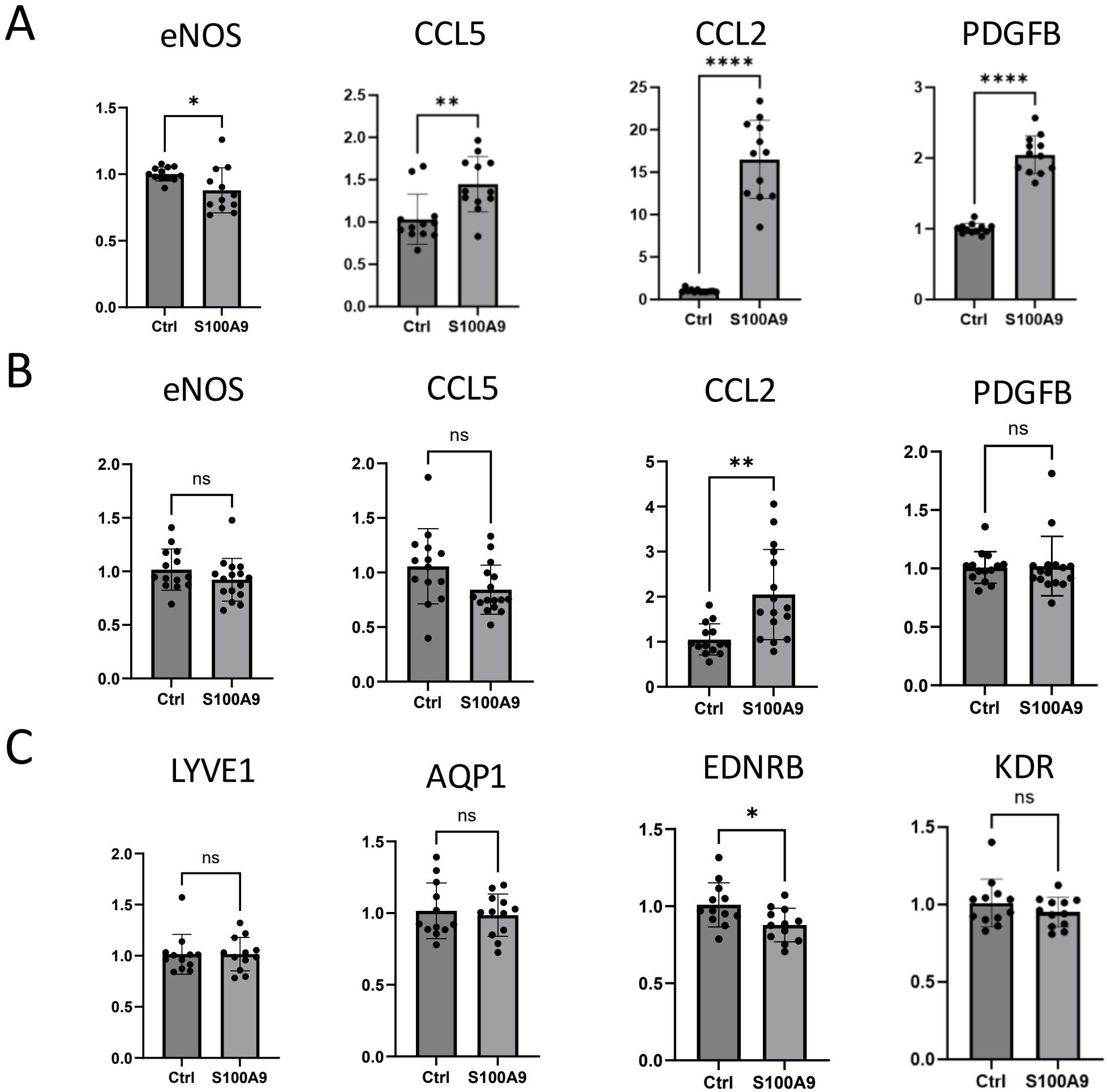

Supplementary Figure 3

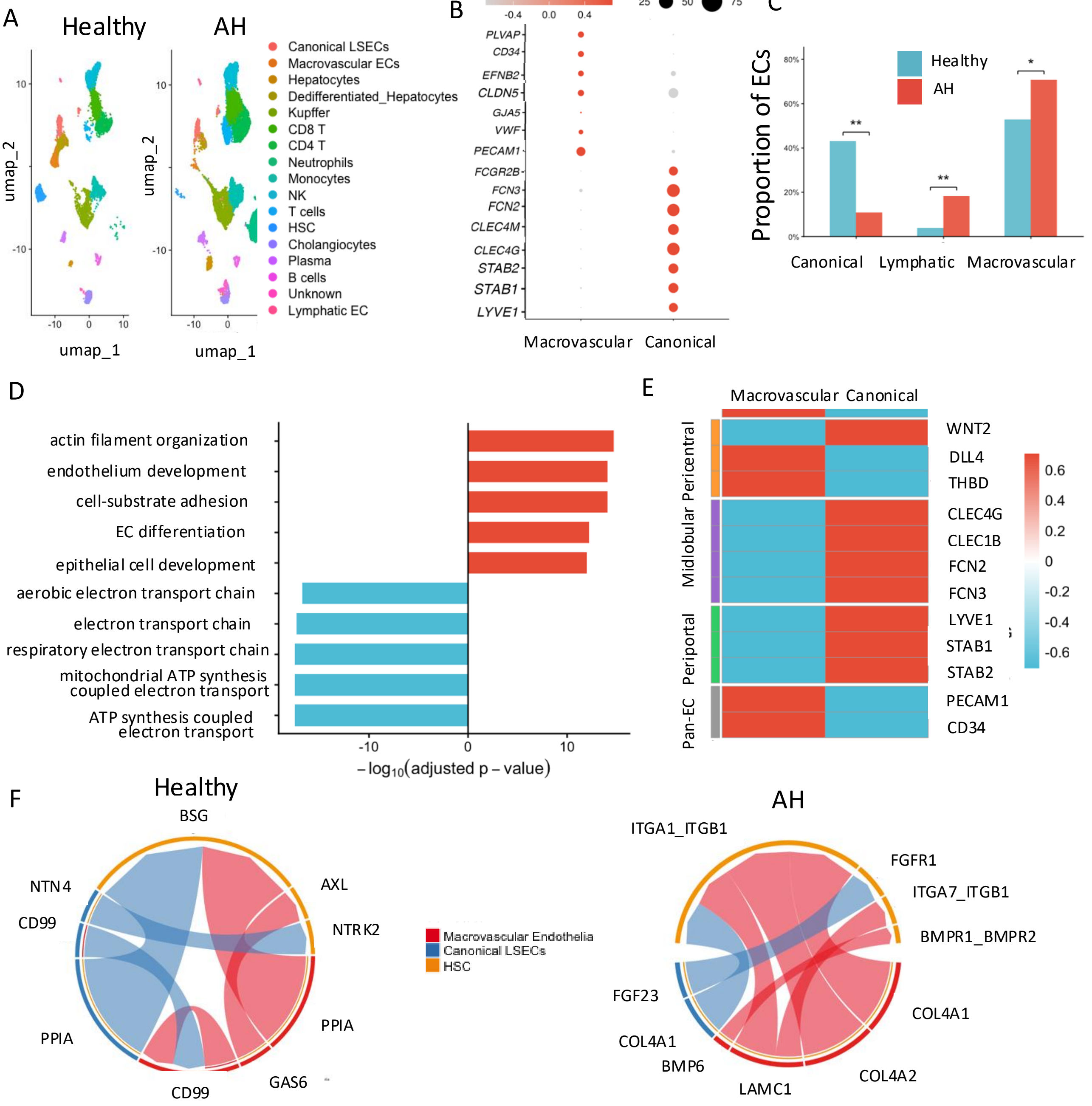

Supplementary Table 1 - RNAseq Patient Information

| Group | Age | Sex | BMI | MELD | Platelets ($10^9$/L) | WBC | Total Bilirubin | INR | Creatinine | Sodium |
|---|---|---|---|---|---|---|---|---|---|---|
| Alcohol Hepatitis | 48.5 (38/57.25) | 4M 4F | 29.6 (24.3/32) | 23.5 (13.8/26.5) | 116 (65.8/320.5) | 6.3 (5/14.3) | 9.1 (3.7/17.8) | 1.7 (1.1/1.9) | 0.6 (0.5/1.1) | 137 (134/139) |
| Healthy Donor | NA | 5M 2F | NA | NA | NA | NA | NA | NA | NA | NA |

NA = Data not available

Supplementary Table 2 - Proteomics Patient Information

| Group | Age | Sex | BMI | MELD | Platelets ($10^9$/L) | WBC | Total Bilirubin | INR | Creatinine | Sodium |
|---|---|---|---|---|---|---|---|---|---|---|
| Alcohol Hepatitis | 40.5 (32.5/ 47.00) | 5 M 5F | 30.9 (25.3/ 39.8) | 28.0 (23.0/ 34.5) | 106.0 (69.8/182.5) | 13.0 (8.4/2 1.6) | 15.6 (11.4/30.9) | 1.7 (1.5/ 2.0) | 0.7 (0.6/2.2) | 132.0 (129.3/1 36.8) |
| Healthy Donor | 30.5 (26.5/ 36.0) | 3M 5F | NA | NA | NA | NA | NA | NA | NA | NA |

NA = Data not available

Supplementary Table 3 - Liver Tissue Patient Information

| Age | Sex | MELD | INR | Plt | AST | ALT | Cr | Bilirubin | BMI |
|---|---|---|---|---|---|---|---|---|---|
| 40 (33/44) | 4M<br>3F | 29 (24/41) | 2.1 (1.7/2.3) | 210 (136/311) | 95 (49/110) | 33 (29/42) | 1.8 (0.9/4.8) | 23.0 (22.9/30.0) | 30.3 (25.2/40.1) |

NA = Data not available

Supplementary Table 4

| Cluster | Gene | Cell Type |
|---|---|---|
| 0 | C1QC, C1QB, FOLR2 | Kupffer cells |
| 1 | CD8B, GZMH | CD8+ cytotoxic T cells |
| 2 | CD40LG, IL7R, CCR6 | CD4+ T cells |
| 3 | FCGR3B, CMTM2 | Neutrophils |
| 4 | VCAN, FCN1, CD300E | Monocytes |
| 5 | KLRF1, CLIC3, SH2D1B | NK cells |
| 6 | THEMIS, INPP4B | T cells (naive/memory) |
| 7 | RGS5, TAGLN, NDUFA4L2 | Hepatic stellate cells (HSCs) |
| 8 | PODXL, SLC9A3R2 | Macrovascular Endothelial Cells (ECs) |
| 9 | NTS, SRPX, CPE | Hepatocytes |
| 10 | 1BICC1, TACSTD2, SFRP5 | Cholangiocytes |
| 11 | CLEC4G, CLEC4M, CLEC1B | Canonical LSECs |
| 12 | IGHA2, IGLC2, IGLC3 | Plasma cells / B cells |
| 13 | FABP1, SAA4, APOC4 | Hepatocytes (de-differentiated) |
| 14 | MS4A1, LINC00926 | B cells |
| 15 | LINC01470, TMEM132C | Unknown |
| 16 | TBX1, CCL21, SEMA3A | Lymphatic endothelial cells |

Supplementary Table 5. Primers for qPCR

| Human Gene Name | Catalog Number | Assay ID |
|---|---|---|
| VCAM1 | 4331182 | Hs0103372_m1 |
| ICAM1 | 4331182 | Hs00164932_m1 |
| CXCL1 | 4331182 | Hs00236937_m1 |
| CXCL2 | 4331182 | Hs00601975_m1 |
| CXCL8 | 4331182 | Hs00174103_m1 |
| IL1B | 4331182 | Hs01555410_m1 |
| TNF | 4331182 | Hs00174128_m1 |
| IL10 | 4331182 | Hs00961622_m1 |
| TGFB | 4331182 | Hs00998133_m1 |
| CD61 | 4331182 | Hs01001469_m1 |
| S100A9 | 4331182 | Hs00610058_m1 |
| STAT1 | 4331182 | Hs01013996_m1 |
| STAT3 | 4331182 | Hs00374280_m1 |
| GAPDH | 4331182 | Hs02786624_g1 |
| eNOS | 4331182 | Hs01574665_m1 |
| CCL5 | 4331182 | Hs00982282_m1 |
| CCL2 | 4331182 | Hs00234140_m1 |
| PDGFB | 4331182 | Hs00966522_m1 |
| LYVE1 | 4331182 | Hs00272659_m1 |

| | | |
|---|---|---|
| AQP1 | 4331182 | Hs01028916_m1 |
| EDNRB | 4331182 | Hs00240747_m1 |
| KDR | 4331182 | Hs00911700_m1 |

**Supplementary Figure Legends**

**Supplementary Figure 1: S100A9 significantly increases expression of inflammatory genes in primary human umbilical vein endothelial cells co-cultured with Thp1 cells.** qPCR of proinflammatory endothelial markers measured in HUVECs in co-culture with Thp1 cells treated with S100A9 (1µg/mL) for 6 hours. 2-tailed unpaired *t* test for 2 groups was used. **p<0.01, ***p<0.001. HUVEC = human umbilical vein endothelial cells. VCAM1 = vascular cell adhesion protein 1, ICAM1 = Intercellular adhesion molecule 1, CXCL1 = CXC motif chemokine ligand 1, CXCL2 = CXC motif chemokine ligand 2, CXCL8 = CXC motif chemokine ligand 8

**Supplementary Figure 2: S100A9 decreased expression of functional markers and increased expression of genes that may induce pathological crosstalk with stellate cells in LSEC co-cultured with Thp1 and primary human monocytes** A) qPCR of LSEC function and metabolic marker and stellate cell crosstalk mediators measured in LSECs in co-culture with Thp1 cells treated with S100A9 (1µg/mL) for 6 hours. B) qPCR of LSEC function and metabolic marker and stellate cell crosstalk mediators measured in LSECs in co-culture with primary human monocytes treated with S100A9 (1µg/mL) for 6 hours. C) qPCR of functional and zonation markers measured in LSECs in co-culture with Thp1 cells treated with S100A9 (1µg/mL) for 6 hours. 2-tailed unpaired *t* test for 2 groups was used. *p<0.05, **p<0.01, ****p<0.0001. eNOS = endothelial nitric oxide synthase, CCL5 = C-C motif chemokine ligand 5, CCL2 = C-C motif chemokine ligand 2, PDGFB = platelet-derived growth factor subunit B, LYVE1 = lymphatic vessel

endothelial hyaluronan receptor 1, AQP1 = aquaporin-1, EDNRB = endothelin receptor type B, KDR = kinase insert domain receptor

**Supplementary Figure 3: Single-cell RNAsequencing reveals alteration of LSEC phenotype in alcohol-asssociated hepatitis**

A) UMAP clustering of liver cell types in healthy and AH liver B) Markers defining macrovascular endothelial cells (ECs) versus canonical LSECs C) Proportions of canonical, lymphatic and macrovascular LSECs in healthy and AH liver D) Pathways up and downregulated in the macrovascular EC cluster in AH E) Differing zonation marker expression between macrovascular ECs and canonical LSECs in AH F) LSEC to hepatic stellate cell signaling in healthy and AH conditions *=FDR<0.05, **=FDR<0.01
